# SNRNP70 regulates the splicing of CD55 to promote osteosarcoma progression

**DOI:** 10.1172/jci.insight.185269

**Published:** 2024-12-20

**Authors:** Wenyue Li, Linzhu Wang, Wen Tian, Weihang Ji, Danyang Bing, Yan Wang, Bingqian Xu, Jiayue Feng, Peng Zhang, Haihai Liang, Yunyan Gu, Baofeng Yang

**Affiliations:** 1 Department of Pharmacy, the First Affiliated Hospital of Zhengzhou University, Zhengzhou, Henan Province, China.; 2 Department of Pharmacology (State-Province Key Laboratories of Biomedicine-Pharmaceutics of China, Key Laboratory of Cardiovascular Research, Ministry of Education), State Key Laboratory of Frigid Zone Cardiovascular Diseases, College of Pharmacy, Harbin Medical University, Heilongjiang Province, China.; 3 Bone and Soft Tissue Sarcoma, The Affiliated Cancer Hospital of Zhengzhou University & Henan Cancer Hospital, Zhengzhou, Henan Province, China.; 4 Department of Nephrology, The Second Affiliated Hospital of Harbin Medical University, Harbin, Heilongjiang Province, China.; 5 Research Unit of Noninfectious Chronic Diseases in Frigid Zone, Chinese Academy of Medical Sciences, Harbin, Heilongjiang Province, China.; 6 Department of Systems Biology, College of Bioinformatics Science and Technology, Harbin Medical University, Harbin, Heilongjiang Province, China.

**Keywords:** Cell biology, Oncology, Molecular biology, Oncogenes

## Abstract

Osteosarcoma (OS) is the most common malignant bone tumor, characterized by a high propensity for metastasis. Recent studies have highlighted the role of alternative splicing in cancer metastasis, although the precise mechanisms underlying aberrant splicing in OS invasion and metastasis remain unclear. Here, we analyzed consistently differentially expressed genes and differentially alternative splicing events between primary and metastatic OS to identify potential genes associated with OS progression. U1 small nuclear ribonucleoprotein 70K (*SNRNP70*) emerged as both differentially expressed and spliced, with elevated *SNRNP70* levels correlating with poor prognosis in pateints with OS. Functional experiments demonstrated that SNRNP70 overexpression enhanced the proliferation and metastasis of OS cells in vitro, while its depletion reduced these capabilities in vivo. Mechanistically, SNRNP70 directly interacted with *CD55*, modulating its alternative splicing and promoting tumor progression in OS. Additionally, metastatic OS samples exhibited increased infiltration of resting immune cells, and single-cell RNA sequencing revealed communication between *SNRNP70*-expressing osteoblastic cells and macrophages via the ADGRE5/CD55 signaling pathway. Overall, our results showed that SNRNP70 knockdown inhibited OS progression, which was associated with the splicing of *CD55*, indicating SNRNP70 as a promising target for OS treatment.

## Abstract 2

SNRNP70 promotes the progression and metastasis of OS through regulating the splicing of CD55.

## Graphical abstract

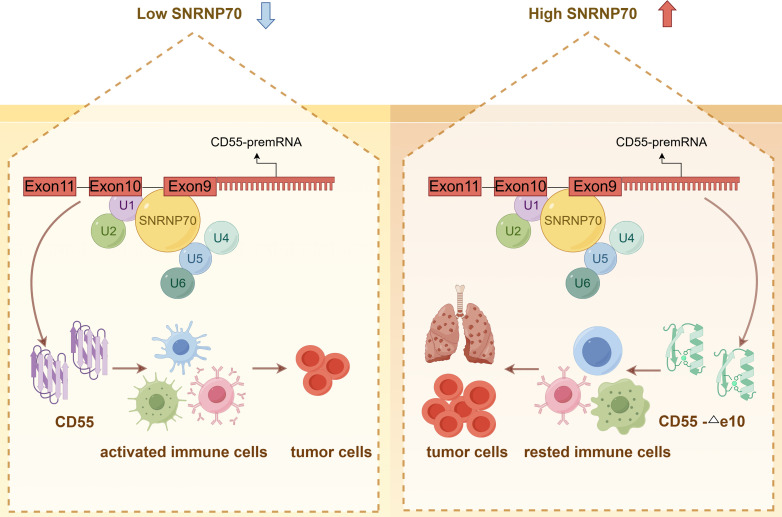

## Introduction

Osteosarcoma (OS) is a highly aggressive malignant bone tumor for which treatment has remained essentially unchanged for more than 30 years (1). Despite the standard treatment regimen of surgery, chemotherapy, and radiation, OS remains associated with a recurrence and metastasis rate exceeding 30%, and the 5-year overall survival rate for patients with metastatic OS is below 20% (2). However, due to the redundant growth signals and an unstable genome, effective molecular therapeutic targets for OS are currently lacking. Therefore, further research into the mechanisms underlying tumor cell metastasis in OS is indispensable.

Alternative splicing (AS) is a finely regulated process wherein non-coding sequences are removed from precursor messenger RNA (pre-mRNA), allowing for the assembly of protein-coding fragments into various isoforms. This process produces proteins with distinct, or even opposing, functions (3). AS is categorized into 5 main types: exon skipping (SE), mutually exclusive exons (MXE), select alternative 5′ splice site (A5SS), retained intron (RI), and alternative 3′ splice site (A3SS). RNA-binding proteins (RBPs) play a critical role in regulating AS, binding to RNA and spliceosome components to control essential cellular functions such as proliferation, DNA repair, immune response, and apoptosis (4–6). Dysregulation of RBPs has been implicated in various diseases, including heart and kidney disorders, as well as cancer (7, 8). For example, *SRSF1* has been shown to regulate the abnormal splicing of *Mcl-1*, inhibiting apoptosis in esophageal squamous tumor cells (9). Consequently, RBPs represent a promising avenue for modulating OS cell proliferation and metastasis through AS.

U1 small nuclear ribonucleoprotein 70K (*SNRNP70*) is an RBP known for its nuclear function, binding to U1 small nuclear RNA to form the U1 snRNP component of the main spliceosome with other subunits (10). Recent evidence indicates that abnormal splicing accompanies the acquisition of invasive properties by OS cells (11). As an essential component of the spliceosome, small nuclear ribonucleoprotein (SNRNP) plays an important role in various cancers, including the regulation of prostate cancer proliferation and cervical cancer cell migration and invasion (12, 13). However, the precise mechanism by which *SNRNP70* influences OS progression via AS remains poorly unclear.

The tumor immune microenvironment (TIME), comprising tumor cells, immune cells, cytokines, and other factors, plays a pivotal role in shaping the immune response to tumors (14). Myeloid cells play a pivotal role within the TIME. The accumulation of tumor-modified myeloid cells is associated with poor outcomes and chemoresistance (15). Recent advancements in single-cell RNA sequencing (scRNA-seq) have enhanced the understanding of cellular diversity and communication within tumors, revealing complex interactions within the TIME. Therefore, there is an urgent need for a thorough exploration of the TIME in OS to identify effective antitumor strategies.

In this study, we identified consistently differentially expressed genes (cDEGs) and differentially alternatively spliced events (DASEs). Among them, *SNRNP70* was identified as a key gene for metastatic OS. Elevated expression of *SNRNP70* was found to positively correlate with the SE of *CD55* in metastatic samples. Further investigation confirmed that SNRNP70 induced an oncogenic splicing switch of CD55-Δe10, contributing to OS progression. Taken together, our work suggests that SNRNP70 may serve as an effective and novel therapeutic target for OS.

## Results

### Transcriptional landscape of primary and metastatic OS.

**Figure 1:**
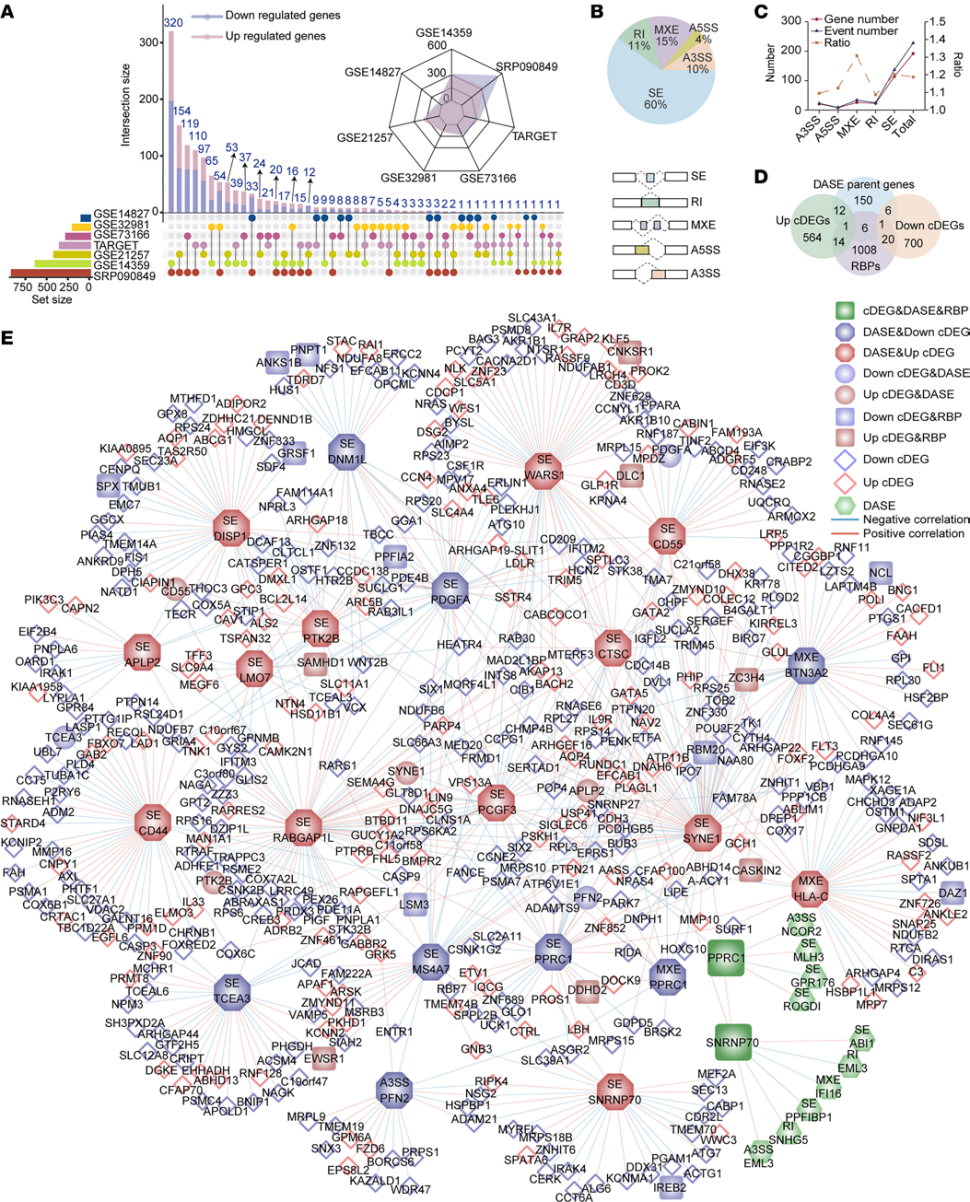
Transcriptional landscape of primary and metastatic OS. (**A**) An UpSet plot displaying intersections of DEGs among 7 datasets in OS (bottom left). A radar chart showing the numbers of cDEGs in the 7 datasets (upper right). Violet color indicates downregulated cDEGs, while pink color indicates upregulated cDEGs. (**B**) A pie chart showing the percentages of DASEs for each type of AS in metastatic tissues compared to primary tissues (top). Five types of AS are depicted in the bottom diagram. (**C**) Multiple line graphs illustrating the numbers of DASEs, DASE parent genes, and the ratio between the two. The left *y* axis represents the number of DASE parent genes and DASEs, while the right *y* axis represents the ratio. The ratios represent the average number of events for one gene in each type of event. (**D**) A Venn diagram displaying the intersection of upregulated or downregulated cDEGs, DASE parent genes, and RBPs. (**E**) Metastatic differential regulatory network between RBPs, DASEs, and cDEGs (Pearson’s correlation analysis). Blue indicates downregulated genes and red indicates upregulated genes in metastatic samples. Regular octagons represent networks constructed using percentage spliced in values of splicing events, while circles and quadrangles represents networks based on gene expression values.

To evaluate gene expression patterns in OS, we identified a total of 1,318 cDEGs across at least 2 datasets comparing primary and metastatic samples (Figure 1A). Among these cDEGs, 591 were upregulated and 727 were downregulated. The cDEGs were enriched in metabolism-associated pathways such as “acylglycerol metabolic process” and “generation of precursor metabolites and energy” (Supplemental Figure 1, A and B; supplemental material available online with this article; https://doi.org/10.1172/jci.insight.185269DS1). In addition to gene expression analysis, we identified 228 DASEs from 176 genes. Among these events, 60% were classified as SE and 15% as MXE (Figure 1B). Metastatic samples showed a bias for skipping within intron regions, but no preference for retention or skipping within exon regions (Supplemental Figure 1C). The parent genes of DASEs were mainly protein-coding genes, with some genes showing multiple splicing events (Figure 1C). Gene Ontology (GO) enrichment analysis revealed that these genes were predominantly involved in cell adhesion and immune related, such as “T cell mediated immunity” (Supplemental Figure 1D). The overlap between cDEGs and DASEs parent genes was approximately 11%. We identified 20 specific genes that exhibited both differential expression and differential splicing. Of these, 13 genes (*PCGF3*, *CD44*, *WARS1*, *HLA-C*, *SNRNP70*, *CTSC*, *LMO7*, *RABGAP1L*, *PTK2B*, *APLP2*, *SYNE1*, *DISP1*, and *CD55*) were upregulated, while 7 genes (*DNM1L*, *PFN2*, *BTN3A2*, *MS4A7*, *TCEA3*, *PPRC1*, and *PDGFA*) were downregulated (Figure 1D and Supplemental Figure 2A). Among these genes, SE events were the most frequent type of AS, with most genes exhibiting only a single type of event (Supplemental Figure 2B). To further explore the role of these splicing events, we constructed a splicing regulatory network for the 20 specific genes. In this network, *PPRC1* and *SNRNP70* were identified as RBPs, while 19 RBPs, 31 DASEs, 171 upregulated cDEGs, and 301 downregulated cDEGs formed 718 regulatory pairs (Figure 1E). Among these, SE_*RABGAP1L*, SE_*SYNE1*, and SE_*WARS1* emerged as the top 3 genes with the highest degree of connectivity in the network. Notably, SE_*SNRNP70* had a degree of 29, highlighting its significant regulatory role (Supplemental Figure 2, C and D).

### SNRNP70 overexpression is associated with metastasis and poor prognosis in OS.

**Figure 2:**
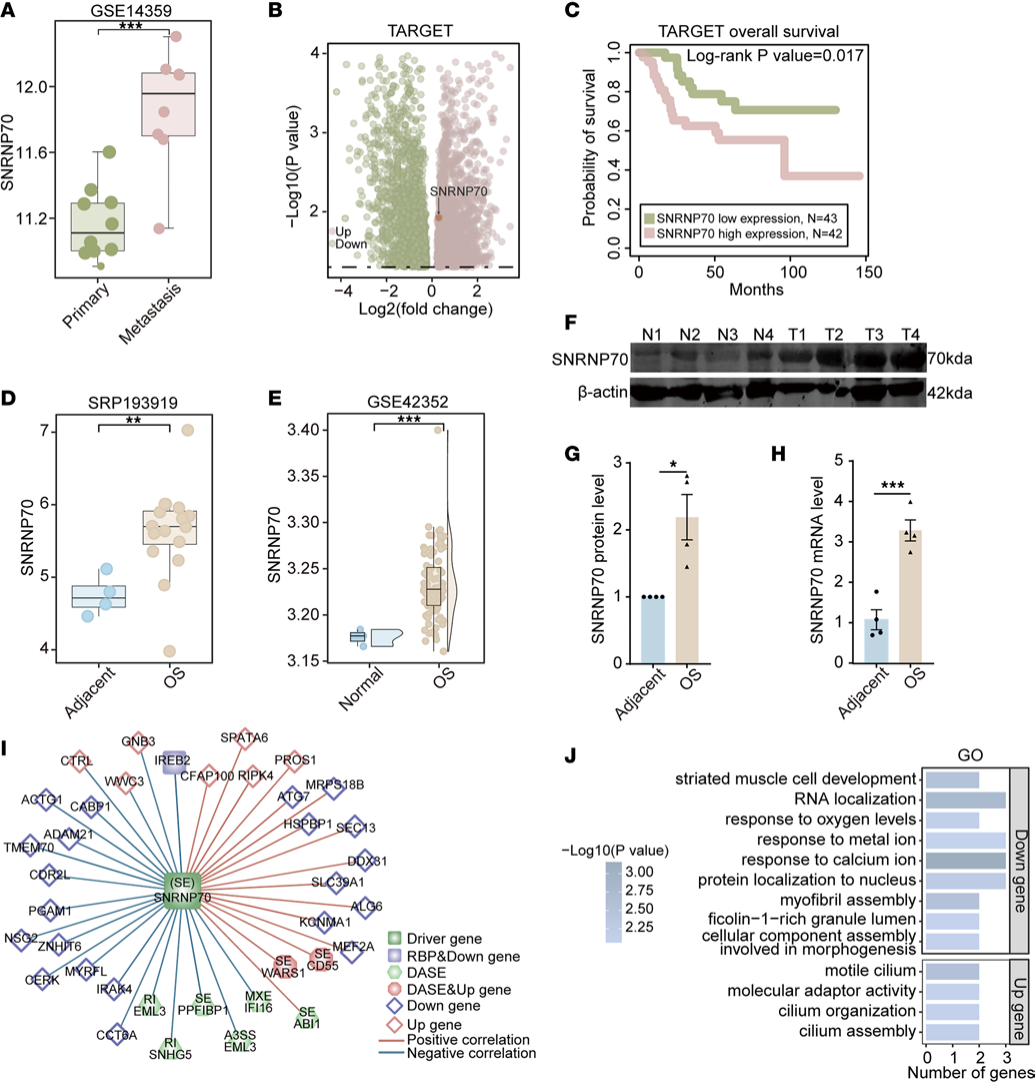
SNRNP70 overexpression is associated with metastasis and poor prognosis in OS. (**A**) Comparison of *SNRNP70* expression levels between metastatic and primary samples in GSE14359. (**B**) Volcano plot illustrating upregulated and downregulated genes between metastasis and primary samples in TARGET. (**C**) Survival analysis demonstrating the effect of *SNRNP70* overexpression on overall survival in TARGET. (**D** and **E**) *SNRNP70* overexpression in OS relative to adjacent tissues in SRP193919 (**D**) and GSE42352 (**E**). (**F**–**H**) Upregulation of SNRNP70 expression at both protein (**F** and **G**) and mRNA (**H**) levels as revealed by WB and qRT-PCR analyses (*n* = 4). (**I**) Splicing regulatory network of *SNRNP70* in the SRP090849 dataset (Pearson’s correlation analysis). (**J**) GO pathway enrichment analysis of cDEGs correlated with *SNRNP70*. Data are presented as mean ± SEM. Statistical significance was calculated using 2-tailed Wilcoxon’s rank-sum test (**A**, **D**, and **E**), DESeq2 method (**B**), log-rank test (**C**), 2-tailed Student’s *t* test (**G** and **H**), or hypergeometric test (**J**). *P < 0.05; **P < 0.01; ***P < 0.001.

*SNRNP70*, as a gene associated with prognosis and encoding RBPs, was identified as a potential regulator of metastasis in OS. Meanwhile, *SNRNP70* was differentially upregulated in metastatic samples compared with primary samples (Figure 2, A and B). Survival analysis showed that elevated *SNRNP70* expression was associated with poorer overall survival and progression-free survival in the Therapeutically Applicable Research to Generate Effective Treatments (TARGET) dataset (Figure 2C and Supplemental Figure 2E). Additionally, *SNRNP70* overexpression was confirmed in OS tumor tissues relative to adjacent non-tumor tissues in both the SRP193919 and GSE42352 datasets (Figure 2, D and E). At both the protein and mRNA levels, SNRNP70 was upregulated in OS samples compared with normal adjacent tissues (Figure 2, F–H).

To better understand the role of SNRNP70, we analyzed its regulatory network derived from the splicing regulatory network. This analysis revealed 37 regulatory pairs involving 7 upregulated cDEGs, 21 downregulated cDEGs, 8 DASEs, and 1 additional RBP (Figure 2I). GO analysis showed that upregulated cDEGs associated with *SNRNP70* were found to be involved in the “motile cilium” pathway, while the downregulated cDEGs were potentially related to signaling pathways, such as “response to calcium ion” and “response to metal ion” (Figure 2J).

### Overexpression of SNRNP70 regulates skipping of exon 10 of CD55.

**Figure 3:**
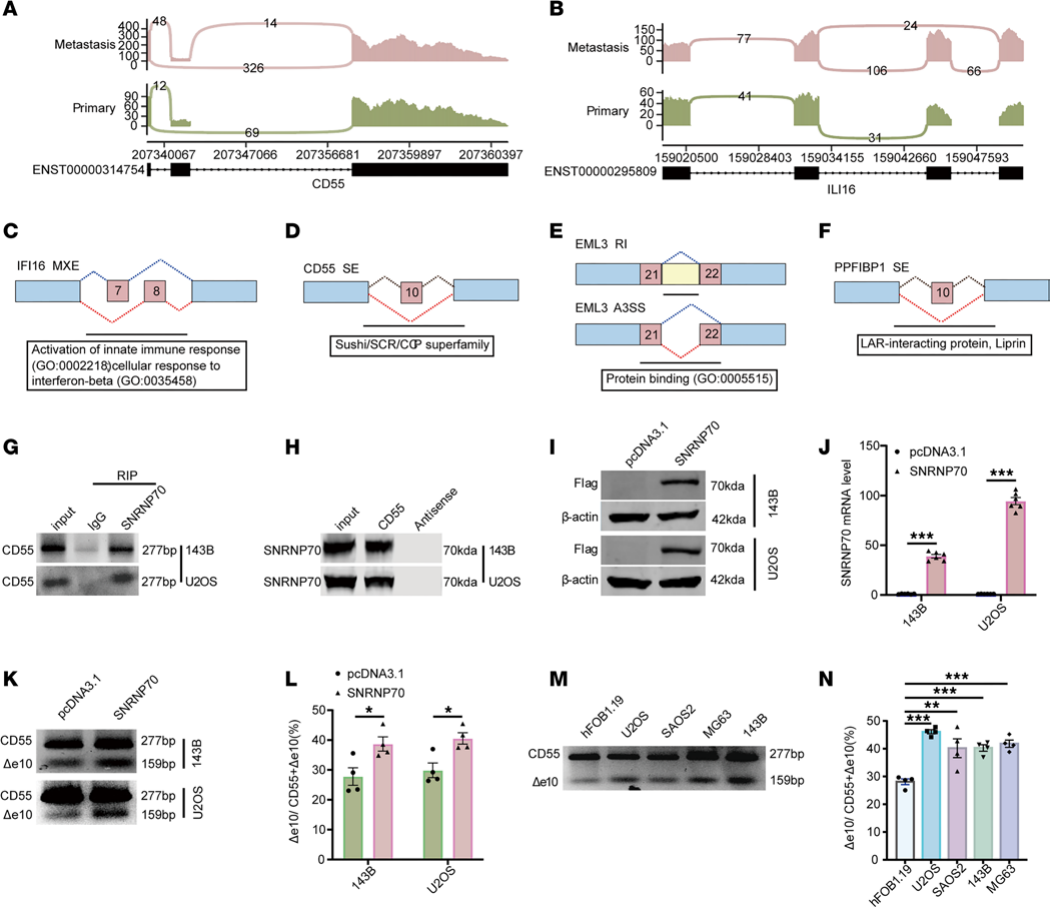
Overexpression of SNRNP70 regulates skipping of exon 10 of CD55. (**A** and **B**) Sashimi plots of *CD55* (**A**) and *ILI16* (**B**) splicing in metastatic and primary tumor samples. (**C**–**F**) Domain function prediction of DASEs correlated with *SNRNP70*. (**G** and **H**) The RIP and RNA pulldown assays showing the interaction between SNRNP70 and *CD55* (*n* = 1). (**I** and **J**) Verification of SNRNP70 overexpression at both protein and mRNA levels relative to the negative control construct (empty pcDNA3.1 plasmid) as revealed by WB (*n* = 4) and qRT-PCR (*n* = 6), respectively. (**K** and **L**) Agarose gel electrophoresis showing the regulation of the AS of CD55 (CD55-Δe10) by SNRNP70 (*n* = 4). (**M** and **N**) qRT-PCR results showing the Δe10/CD55+Δe10 values in OS and normal bone cell lines (*n* = 4). Data are presented as mean ± SEM. Statistical significance was calculated using a 2-tailed Student’s *t* test (**J** and **L**) or 1-way ANOVA followed by Dunnett’s multiple-comparison test (**N**). *P < 0.05; **P < 0.01; ***P < 0.001.

*SNRNP70* was found to be positively correlated with the splicing of *CD55*, *WARS1*, and *ABI1*, while negatively correlated with the splicing of *EML3*, *SNHG5*, *PPFIBP1*, and *IFI16* (Figure 2I). Specifically, we observed the skipping of exon 10 in *CD55* pre-mRNA, the inclusion of exon 10 in *PPFIBP1* pre-mRNA, mutually exclusive exons 7 and 8 in *IFI16* pre-mRNA, inclusion of an intron between exons 21 and 22 in *EML3* pre-mRNA, and alternative 3′ splice site (A3SS) events involving exons 21 and 22 in *EML3* during metastasis (Figure 3, A and B, and Supplemental Figure 2F). InterPro indicated that these splicing sequences were enriched in the complement control protein (CCP) superfamily, leukocyte common antigen–related–interacting (LAR-interacting) protein, protein binding, activation of innate immune response, etc. (Figure 3, C–F).

Using RBPmap, we predicted binding sites between *SNRNP70* and *CD55*, which were experimentally validated through RNA immunoprecipitation (RIP) and RNA pulldown assays (Figure 3, G and H). Following transfection of 143B cells with a plasmid overexpressing *SNRNP70*, both the protein and mRNA levels of SNRNP70 were upregulated successfully (Figure 3, I and J). Notably, agarose gel electrophoresis results demonstrated that SNRNP70 played a key role in regulating the splicing of *CD55* (Figure 3, K and L). Further analysis demonstrated that the CD55-Δe10 isoform was more abundantly expressed in OS cell lines (U2OS, SAOS2, MG63, 143B) compared with a normal bone cell line (hFOB1.19) (Figure 3, M and N).

### CD55-Δe10 is crucial for the tumorigenicity of OS cells in vitro.

**Figure 4:**
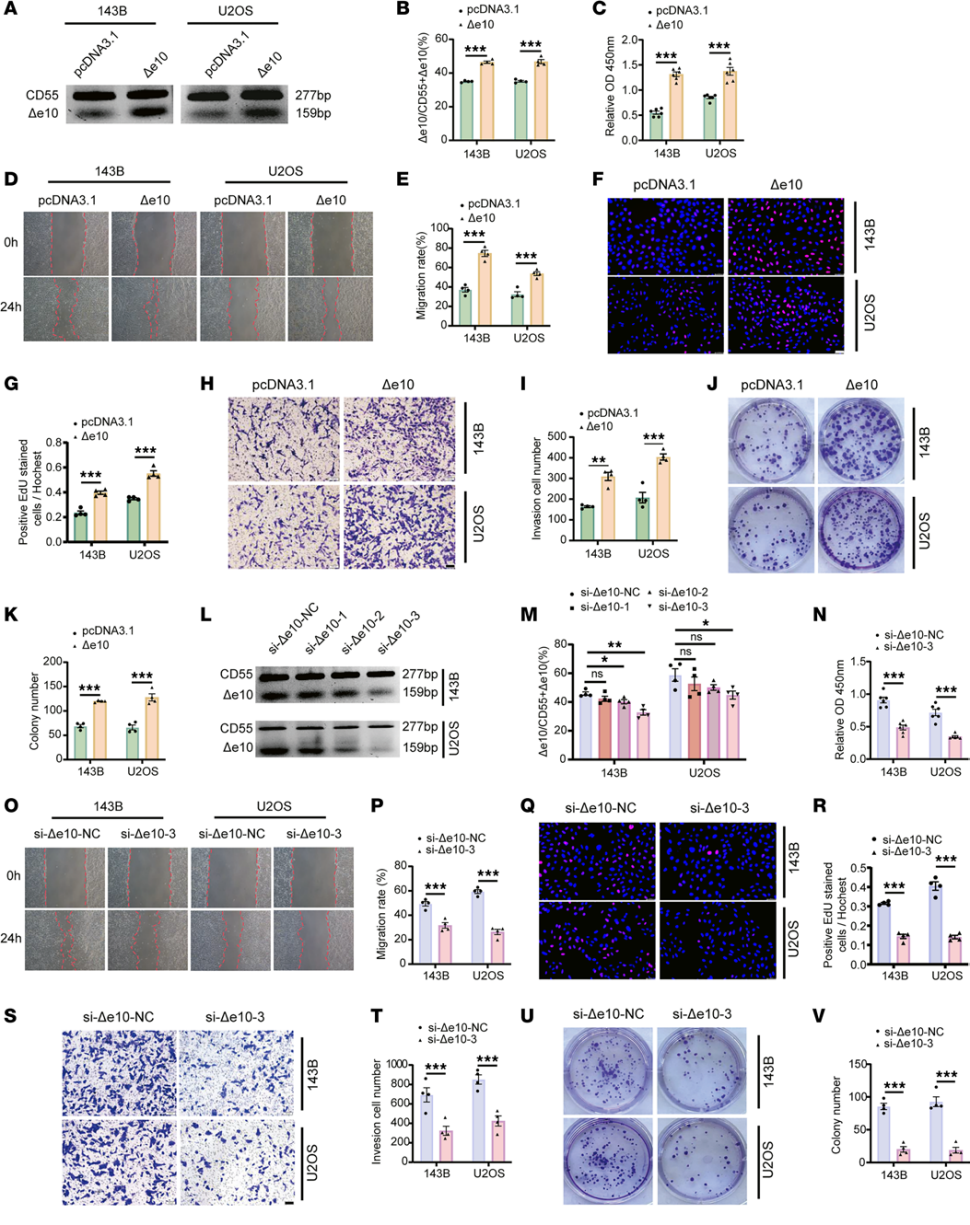
CD55-Δe10 is crucial for the tumorigenicity of OS cells in vitro. (**A** and **B**) qRT-PCR analysis the efficiency of CD55-Δe10 overexpression in OS cells (*n* = 4). (**C**) The CCK-8 assay showing that CD55-Δe10 substantially enhanced cell viability (*n* = 6). (**D** and **E**) The wound-healing assay depicting the promoting effect of CD55-Δe10 overexpression on the migration (original magnification, × 40; *n* = 4). (**F** and **G**) The EdU assay showing that overexpressing CD55-Δe10 increased cell proliferation (scale bar: 50 μm; *n* = 4). (**H** and **I**) The Transwell assay showing the promoting effect of CD55-Δe10 overexpression on the invasiveness (scale bar: 100 μm; *n* = 4). (**J** and **K**) The colony formation assay illustrating the facilitating effect of CD55-Δe10 overexpression on the colony-forming capacity (*n* = 4). (**L** and **M**) qRT-PCR analysis the efficiency of CD55-Δe10 knockdown in OS cells (*n* = 4). (**N**) The CCK-8 assay for assessing the impact of CD55-Δe10 silencing on cell viability (*n* = 4). (**O** and **P**) The wound-healing assay to elucidate the inhibitory effects of silencing CD55-Δe10 on the migration (original magnification, × 40; *n* = 4). (**Q** and **R**) The EdU assay evaluating the cell proliferation after CD55-Δe10 knockdown (scale bar: 50 μm; *n* = 4). (**S** and **T**) The Transwell assay to assess the inhibitory effects of silencing CD55-Δe10 on the invasiveness (scale bar: 100 μm; *n* = 4). (**U** and **V**) The colony formation assay to evaluate the inhibitory effects of CD55-Δe10 knockdown on the colony-forming capacity (*n* = 4). Data are presented as mean ± SEM. Statistical significance was calculated using a 2-tailed Student’s *t* test (**B**, **C**, **E**, **G**, **I**, **K**, **M**, **N**, **P**, **R**, **T**, and **V**) or 1-way ANOVA followed by Dunnett’s multiple-comparison test (**M**). *P < 0.05; **P < 0.01; ***P < 0.001.

To investigate the role of CD55-Δe10 in OS, we first verified the successful upregulation of CD55-Δe10 expression in OS cells (Figure 4, A and B). Subsequently, we evaluated the impact of CD55-Δe10 overexpression on OS cell behavior. Our results demonstrated that upregulation of CD55-Δe10 enhanced the viability and migration ability of U2OS and 143B cells (Figure 4, C–E). Moreover, both ethynyl-2′-deoxyuridine (EdU) and Transwell assays revealed increased proliferation and invasion ability of cells with stable overexpression CD55-Δe10 compared with control cells (Figure 4, F–I). Furthermore, cells stably overexpressing CD55-Δe10 formed larger and more colonies than control cells (Figure 4, J and K). Conversely, knocking down CD55-Δe10 using siRNAs (Figure 4, L and M) led to decrease in cell viability and migration (Figure 4, N–P). The proliferative and invasive abilities of cells with CD55-Δe10 knockdown were also impaired, as evidenced by the EdU and Transwell assays (Figure 4, Q–T). Additionally, a colony formation assay showed that CD55-Δe10 knockdown resulted in smaller and fewer colonies compared with control cells (Figure 4, U and V).

### CD55-Δe10 knockdown partially reverses the oncogenic function of SNRNP70 overexpression.

**Figure 5:**
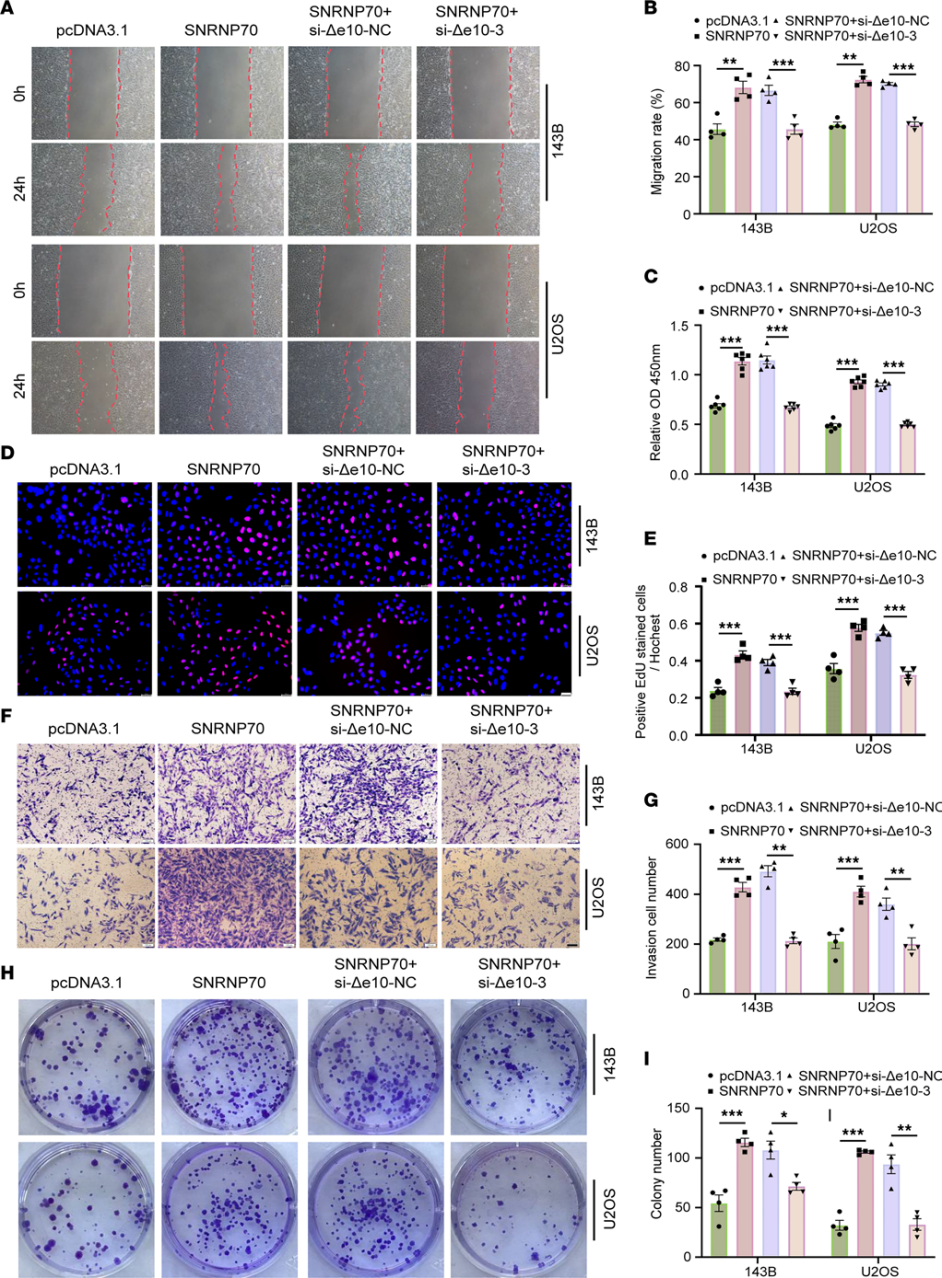
CD55-Δe10 knockdown partially reverses the oncogenic function of SNRNP70 overexpression. (**A** and **B**) The migration of the OS cells was determined by wound-healing assay (original magnification, ×4 0; *n* = 4). (**C**–**E**) Cell viability and proliferation were examined by CCK-8 (*n* = 6) and EdU (scale bar: 50 μm; *n* = 4) assays. (**F** and **G**) Transwell assays in which the numbers of invaded cells were quantified (scale bar: 100 μm; *n* = 4). (**H** and **I**) The colony formation assay was performed to illustrate the colony-forming capacity (*n* = 4). Data are presented as mean ± SEM. Statistical significance was calculated using 1-way ANOVA followed by Tukey’s multiple-comparison test (**B**, **C**, **E**, **G** and **I**). *P < 0.05; **P < 0.01; ***P < 0.001.

To evaluate the impact of CD55-Δe10 on the oncogenic effects of SNRNP70 overexpression, we transfected CD55-Δe10–knockdown cells with an SNRNP70 overexpression construct. The results showed that migration of cells overexpressing SNRNP70 was increased compared with control cells (Figure 5, A and B). Moreover, overexpression of SNRNP70 enhanced cell viability and proliferation in both 143B and U2OS cells (Figure 5, C–E). The invasive capability and colony formation ability of OS cells overexpressing SNRNP70 were also improved compared with control cells (Figure 5, F–I). Notably, CD55-Δe10 knockdown was found to inhibit the migration, viability, proliferation, invasion, and colony formation abilities of cells with SNRNP70 overexpression in 143B and U2OS cells (Figure 5, A–I). Collectively, these findings suggest that knockdown of CD55-Δe10 partially reverses the oncogenic effects of SNRNP70 overexpression, impacting the growth and migration of OS cells.

### Knockdown of SNRNP70 inhibits OS progression and metastasis.

**Figure 6:**
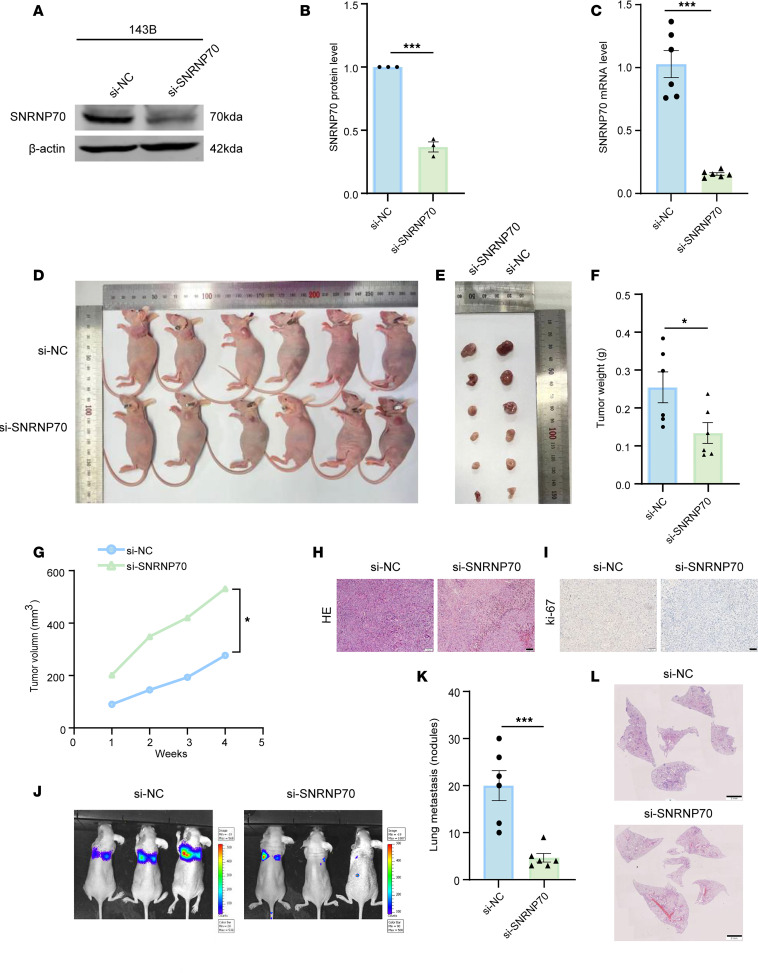
Knockdown of SNRNP70 inhibits tumor progression and metastasis. (**A**–**C**) Verification of SNRNP70 silencing efficiency by siSNRNP70 as evidenced by the WB (*n* = 3) and qRT-PCR (*n* = 6) analyses. (**D**) BALB/c nude mice were implanted with siNC-pretreated or siSNRNP70-pretreated 143B cells. After 4 weeks, the mice were sacrificed (*n* = 6 for each group). (**E**) Tumor tissues dissected from mice 4 weeks after transplantation. (**F**) Tumor weight measured at 4 weeks after implantation. (**G**) Tumor size measured every week after implantation. (**H** and **I**) H&E and IHC staining of Ki-67 in different tumor tissues (scale bar: 50 μm). (**J**–**L**) Representative IVIS imaging (**J**), the number of lung metastatic nodules (**K**), and H&E for lung sections (**L**) from mice tail injected with the controlled and SNRNP70-knockout 143B cells; *n* = 6 animals for each group. Data are presented as mean ± SEM. Statistical significance was calculated using a 2-tailed Student’s *t* test (**B**, **C**, **F**, **G**, and **K**). *P < 0.05; ***P < 0.001.

To evaluate the impact of SNRNP70 on OS tumorigenicity and metastasis, 143B cells were transfected with either SNRNP70 siRNA (siSNRNP70) or negative control siRNA (siNC) for SNRNP70 silencing (Figure 6, A–C). These cells were then subcutaneously injected into BALB/c nude mice to assess their tumorigenicity in vivo (Figure 6, D and E). The results showed that both the average weight and tumor volume were reduced in SNRNP70-knockdown mice compared with the siNC group (Figure 6, F and G). Additionally, histological analysis of tumor sections stained with hematoxylin and eosin (H&E) revealed the pathological state. Representative immunohistochemistry (IHC) staining images showed a decrease in the number of Ki-67^+^ cells in SNRNP70-knockdown mice compared with the siNC group (Figure 6, H and I). To further investigate the role of SNRNP70 in lung metastasis, we established a mouse model of lung metastasis through tail vein injection. The extent of lung metastasis was diminished in SNRNP70-knockdown mice compared with the siNC group (Figure 6J). H&E staining revealed smaller and fewer numbers of nodules in SNRNP70-knockdown mice relative to siNC-treated mice (Figure 6, K and L). These findings demonstrate that SNRNP70 knockdown suppresses both tumor growth and metastasis of OS in vivo.

### Metastatic OS exhibits a notable infiltration of quiescent immune cells.

**Figure 7:**
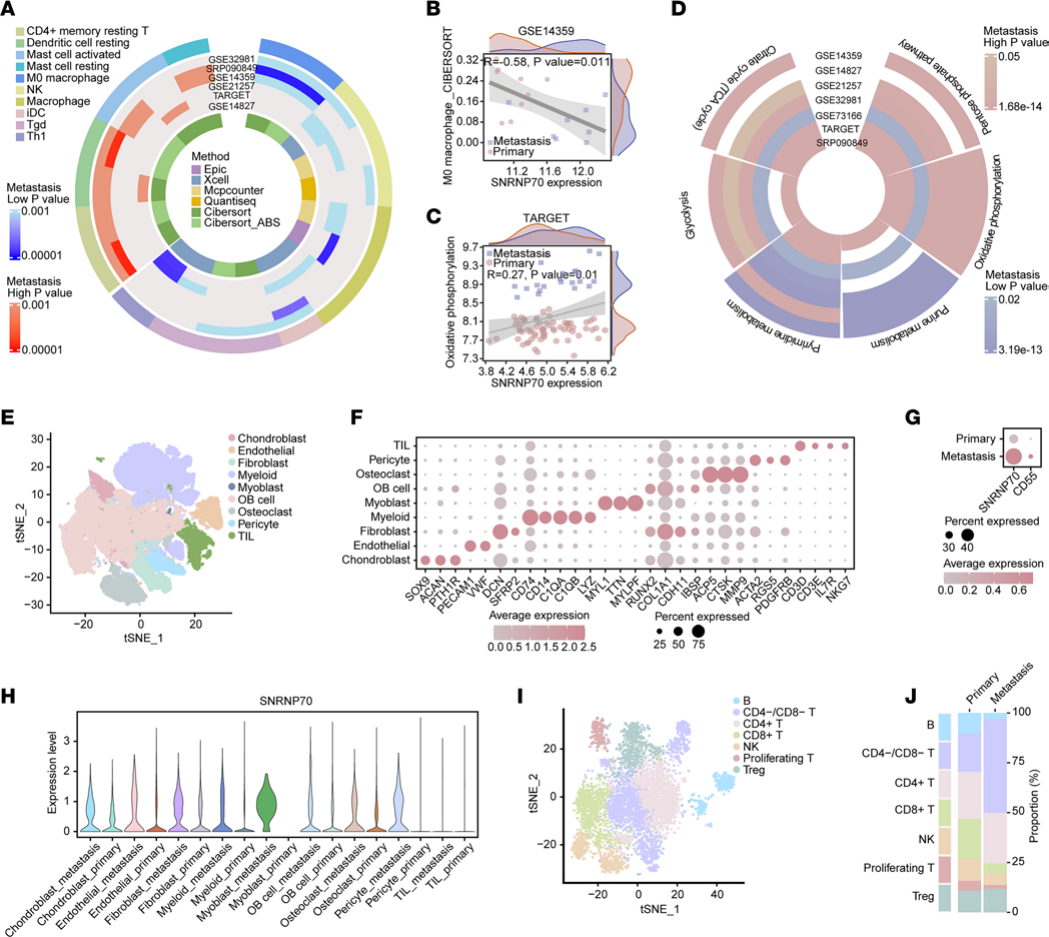
Metastatic OS exhibits high infiltration of quiescent immune cells. (**A**) Comparison of immune cell infiltration between primary and metastatic samples across 7 datasets. The innermost ring displays color variation according to methods, while the middle layer presents a heatmap of *P* values, with darker shades indicating higher significance. Red denotes cells with high infiltration proportions in metastatic samples, while blue represents cells with low infiltration proportions. Different colors represent different immune cells in the outermost layer. NK, natural killer cell; Tgd, γδ T cells; Th1; T helper 1 cells. (**B**) Pearson’s correlation analysis illustrating the relationship between *SNRNP70* expression and infiltration proportions of M0 macrophages in GSE14359. (**C**) Pearson’s correlation analysis demonstrating the association between *SNRNP70* expression and activities of the oxidative phosphorylation pathway in GSE14359. The *x* axis represents *SNRNP70* expression values, while the *y* axis represents immune cell infiltration or metabolic pathway scores. The top depicts the distribution of expressed values, while the right illustrates the distribution of scores in 2 types. (**D**) Heatmap depicting metabolic pathway activities between primary and metastasis samples across 7 datasets. (**E**) t-SNE visualization of 91,430 cells analyzed by scRNA-seq and integrated across 9 OS samples. Clusters are annotated for cell types using canonical markers and color-coded accordingly. (**F**) Dot plot displaying the expression of signature genes across the 9 clusters. The size of dots represents the proportion of cells expressing the particular marker, while the spectrum of color indicates the mean expression levels of the markers. (**G**) Dot plot demonstrating *SNRNP70* and *CD55* expression in GSE152048. (**H**) Violin plot showing *SNRNP70* expression across the 9 clusters between primary and metastatic tissues. (**I**) t-SNE visualization of 7 subsets of TILs. (**J**) Comparison of the proportions of subsets of TILs between primary and metastatic tissues. Statistical significance was calculated using a 2-tailed Wilcoxon’s rank-sum test (**A** and **D**).

The enrichment results from the GO analysis motivated us to explore the heterogeneity of immune cell infiltration and metabolic pathways between primary and metastatic OS samples. In metastatic OS, a significant proportion of immune cells were quiescent, including CD4^+^ memory cells, dendritic cells, and mast cells. In contrast, proinflammatory immune cells such as natural killer cells, γδ T cells, and T helper 1 cells were present in lower proportions (Figure 7A). Correlation analysis revealed that high expression of *SNRNP70* was correlated with reduced infiltration of M0 macrophages (Figure 7B and Supplemental Figure 3A). Metabolic pathway activities varied across 7 datasets between primary and metastatic samples. In the 2 datasets where *SNRNP70* was upregulated in metastatic samples, pathways including the tricarboxylic acid cycle, oxidative phosphorylation, and the pentose phosphate pathway were activated (Figure 7D). Furthermore, the expression level of *SNRNP70* was positively associated with the activities of oxidative phosphorylation, tricarboxylic acid cycle, and pentose phosphate pathways, while negatively correlated with the purine metabolism pathway (Figure 7C and Supplemental Figure 3, B–E). Furthermore, 4 downregulated cDEGs associated with *SNRNP70* were linked to the hydrogen ion network, and 3 were connected to the adenosine triphosphate (ATP) network (Supplemental Figure 3F).

### SNRNP70-positive osteoblastic cells establish communication with macrophages via ADGRE5/CD55 signaling.

To explore the immune cell landscape in primary and metastatic OS, we analyzed an scRNA-seq dataset from GSE152048. Dimensionality reduction and clustering of 91,430 cells from OS samples resulted in 9 distinct groups based on established markers (Figure 7, E and F). Visualization using *t*-distributed stochastic neighbor embedding (t-SNE) displayed a well-distributed population of cells in the integrated data (Supplemental Figure 4A). Metastatic cells exhibited elevated expression of *SNRNP70*, which was prevalent across metastatic clusters except for tumor-infiltrating lymphocytes (TILs) (Figure 7, G and H), whereas *CD55* showed low expression in all cells (Figure 7G and Supplemental Figure 4B). Metastatic samples demonstrated higher proportions of osteoblastic (OB) cells and TILs, and lower ratios of myeloid cells and osteoclasts (Supplemental Figure 4, C and D). To delineate the heterogeneity of TILs, unsupervised clustering of TILs revealed 12 clusters, which were categorized into 7 common TIL subsets based on marker gene expression (Figure 7I and Supplemental Figure 4, E and F). Compared with primary OS, metastatic samples had a higher proportion of CD4^–^ and CD8^–^ T cells and a lower proportion of CD8^+^ T cells (Figure 7J). Both *SNRNP70*^+^ and *SNRNP70*^–^ OB cells sent signals of similar intensity to immune subsets; however, *SNRNP70*^+^ OB cells received more signals from immune subsets (Supplemental Figure 4, G and H).

**Figure 8:**
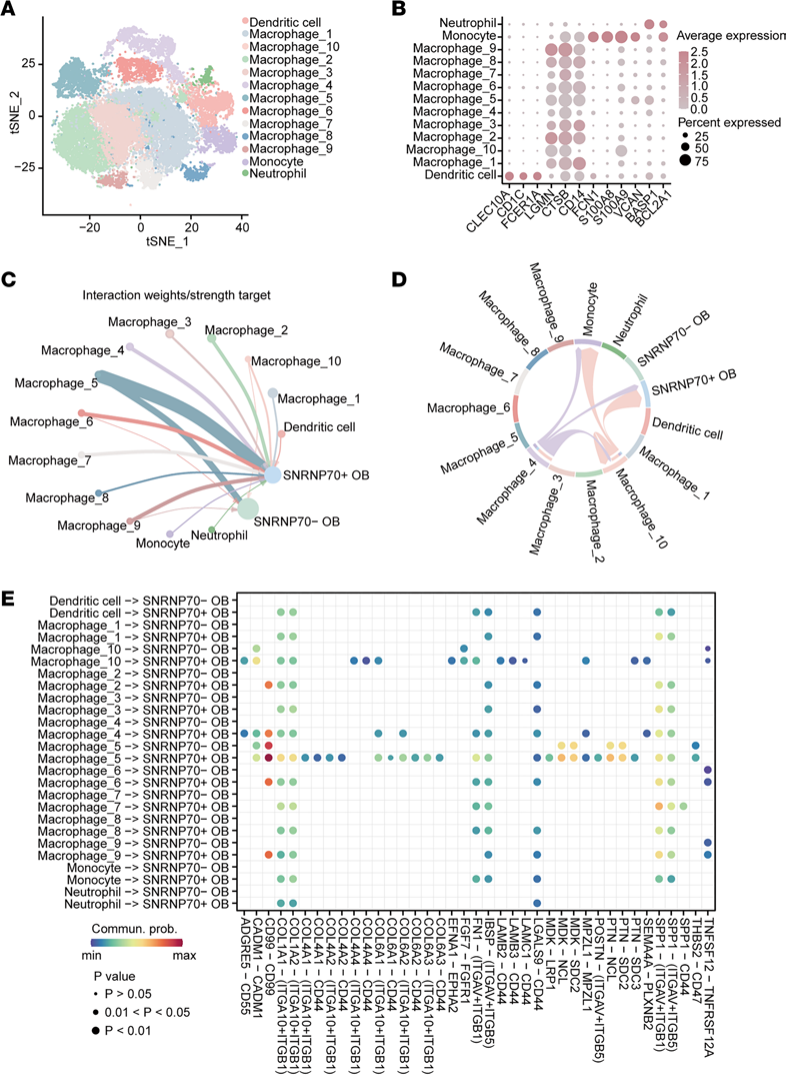
*SNRNP70*^+^ OB cells communicate with macrophages via ADGRE5/CD55 signaling. (**A**) t-SNE visualization of subsets of myeloid cells. (**B**) Dot plot displaying the expression of signature genes across the 13 subsets. (**C**) Visualization and analysis of cell-cell communication using CellChat. Circle plot depicting interaction weights from myeloid cells to *SNRNP70*^+^ OB cells or *SNRNP70*^–^ OB cells. (**D**) Predicted cell-cell interactions via ADGRE5/CD55 signaling. (**E**) Interacted ligand-receptor pairs from myeloid cells to *SNRNP70*^+^ OB cells or *SNRNP70*^–^ OB cells.

Further analysis of myeloid cells identified 13 clusters, including dendritic cells, monocytes, neutrophils, and 10 macrophage clusters (Figure 8, A and B). CellChat analysis revealed notable communication from myeloid cell subsets to *SNRNP70*^+^ OB cells, with no bias in signaling from *SNRNP70*^+^ and *SNRNP70*^–^ OB cells to myeloid subsets (Figure 8C). Specifically, we identified ADGRE5/CD55 signaling originating from macrophage 4 toward *SNRNP70*^+^ OB cells, monocytes, and macrophage 10 (Figure 8D). Additionally, the SPP1 signaling pathway was involved in communication from myeloid cells to *SNRNP70*^+^ OB cells, where *SPP1* interacted with *ITGAV*, *ITGB1*, and *ITGB5* (Figure 8E).

## Discussion

In this study, we revealed that *SNRNP70* was upregulated in metastatic OS through integrating gene expression and splicing profiling. Furthermore, overexpression of SNRNP70 was found to enhance OS cell viability, migration, and invasion by influencing the AS of *CD55* both in vivo and in vitro. Additionally, there was frequent crosstalk observed between *SNRNP70*^+^ OB cells and immune cells. Collectively, our findings indicate that SNRNP70 could serve as a promising therapeutic target in OS.

By integrating cDEGs and DASEs between primary and metastatic samples, we identified 20 genes exhibiting both differential expression and splicing patterns. Changes in these oncogenes and tumor suppressor genes were associated with various aspects of OS progression, including proliferation, migration, and chemoresistance (16, 17). Additionally, switching of splicing isoforms was found to play a role in regulating key processes in tumor cell biology, including cell cycle, ferroptosis, and immune evasion in OS (18, 19). Given the significance of abnormal gene expression levels and splicing isoforms in OS, it was hypothesized that genes displaying both differential expression and splicing alterations could be potential targets for OS treatment. Notably, some of these genes, like *LMO7* and *CD44*, had been previously implicated in cancer. *LMO7* was involved in tumor-intrinsic innate immunity and immune evasion, while *CD44* was associated with cancer stem cells and exhibits spliced variants that contribute to cancer progression (20, 21).

Among the identified genes, *SNRNP70*, an RBP, was notably upregulated in OS and correlated with poor prognosis. While previous studies have linked *SNRNP70* to diseases like myotonic dystrophy and rheumatoid arthritis (22, 23), its role in oncogenesis had not been explored until now. Our study confirmed that SNRNP70 was upregulated in OS tissues and its knockdown inhibited OS cell proliferation and metastasis both in vitro and in vivo.

We demonstrated that SNRNP70 regulates the skipping of exon 10 of *CD55*, thereby promoting OS metastasis. RBPs like SNRNP70 play a crucial role in posttranscriptional gene regulation by influencing RNA splicing, stability, and translation (24). Dysregulation of RNA splicing could lead to the production of abnormal protein isoforms, contributing to tumor development and metastasis (25, 26). Given the importance of splicing in cancer, we focused on DASEs regulated by *SNRNP70*. Our study confirmed that SNRNP70 positively correlates with *CD55* exon 10 skipping in metastatic samples and validated this interaction through RIP and RNA pulldown assays. It was reported that CD55 was overexpressed in OS tissue compared with normal tissue, indicating its involvement in progression of OS (27). Meanwhile, SNRNP70’s ability to regulate the AS of *CD55* was further verified by agarose gel electrophoresis assay. *CD55*, a decay-accelerating factor, protected cells from complement-mediated attack and was involved in cancer progression through mechanisms like cell survival, angiogenesis, and apoptosis inhibition (28–30). Our findings suggest that the expression of CD55-Δe10 affects OS cell growth and migration, and its knockdown can partially reverse the oncogenic effects of SNRNP70 overexpression, highlighting SNRNP70’s role in promoting OS cell proliferation and metastasis through *CD55* splicing regulation.

*SNRNP70* may contribute to OS metastasis by impacting TIME and metabolic pathways. Growing evidence indicates that TIME and metabolic reprogramming can boost cancer cell immune evasion, survival, and metastasis (31). Within myeloid lineage cells, macrophages play diverse roles in various cancers (32). We found metastatic OS samples exhibited high infiltration of resting immune cells and altered metabolic activity. The negative correlation between *SNRNP70* and M0 macrophages was coupled with increased interactions between *SNRNP70*^+^ OB cells and immune subsets. Emerging studies indicates that signals from T cells and macrophages are linked to tumor growth and metastasis (33), implying that *SNRNP70*^+^ OB cells might enhance metastasis through communication with T cells and macrophages in OS. In addition, metastatic OS samples were characterized by high enrichment scores of metabolic pathways, supporting the notion of increased metabolic activity in metastatic cancer cells (34). The expression level of *SNRNP70* was positively correlated with the enrichment scores of the tricarboxylic acid cycle and oxidative phosphorylation pathways, whose upregulation has been shown to promote tumor metastasis.

Despite these insights, our study has limitations, including a relatively small number of clinical samples. Future research should involve larger sample sizes to validate these findings. Additionally, the impact of SNRNP70 on other spliced target genes and its specific effects on immune cell status warrant further investigation. Continuing research into the relationship between SNRNP70, AS, and OS can pave the way for novel therapeutic approaches for patients with OS.

## Methods

### Sex as a biological variable.

Sex was not considered as a biological variable. For in vivo studies, only female mice were used based on practical considerations.

### Data acquisition of OS samples.

Transcriptome data of primary and metastatic OS samples were retrieved from the NCBI Sequence Read Archive (SRA, https://www.ncbi.nlm.nih.gov/sra/), Gene Expression Omnibus (GEO, http://www.ncbi.nlm.nih.gov/geo/), and TARGET (https://ocg.cancer.gov/ programs/target/). Supplemental Table 1 shows the statistics of OS samples. For SRP193919 and SRP090849 datasets, all fastq files were aligned to the human reference genome build GRCh38 with HISAT (https://daehwankimlab.github.io/hisat2/download/). The transcript quantification tool featureCounts v2.0.1 (https://sourceforge.net/projects/subread/files/subread-2.0.1/subread-2.0.1-Linux-x86_64.tar.gz/download) was then used to generate integer-based read counts. The gene expression levels were represented by the expected number of fragments per kilobase of transcript sequence per million base pairs sequenced.

### Identification of cDEGs and DASEs between primary and metastatic samples.

The expression profiles of the 5 datasets, including GSE14359, GSE14827, GSE21257, GSE32981, and GSE73166, were log_2_ transformed, and statistical significance (between primary and metastatic samples) was assessed using a Student’s *t* test. We performed differential expression analysis using the R package DESeq2 (v1.34.0) on RNA-seq data comparing primary and metastatic samples from both TARGET and SRP090849 (35). A *P* value of less than 0.05 was considered statistically significant. We retained the cDEGs identified in at least 2 datasets.

Python rMATS (v4.1.1) was employed to analyze DASEs between primary and metastatic samples in the SRP090849 dataset, and package “ggsashimi” was used to visualize splicing events (36,37). A false discovery rate (FDR) of less than 0.05 was considered statistically significant. The RBPmap database was used to predict potential binding regions between RBPs and mRNAs (38).

### Functional enrichment and survival analysis.

Functional enrichment analysis of genes was performed using R packages “clusterProfiler” (v4.2.0) and “DOSE” (v3.20.0). *P* value was adjusted using the Benjamini-Hochberg (BH) procedure. Kaplan-Meier curves for 2 types of patients were generated using the R package “survival” (v3.3.1).

### Construction of splicing regulatory network.

The RBP gene set was obtained from the RBPDB database (39). Differentially expressed RBPs, DASEs and cDEGs were selected to construct the splicing regulatory network in the SRP090849 dataset. Pearson’s correlation analysis was employed to analyze the correlation between differentially expressed RBPs and DASEs, as well as the correlation between DASEs and cDEGs. The splicing regulatory network was considered significant based on the following criteria: (a) *P* value less than 0.05 in metastatic samples, (b) *P* value greater than 0.05 in primary samples, (c) and |*r*| greater than 0.8 in metastatic samples. Cytoscape software (v3.7.2, https://cytoscape.org/) was used to visualize the splicing regulatory network.

### Immune infiltration analysis.

The proportion of infiltrating immune cells was estimated across 7 datasets using transcriptome-based algorithms, including CIBERSORT, EPIC, TIMER, QUANTISEQ, MCPCOUNTER, CIBERSORT-ABS, and XCELL. These algorithms are available on the websites CIBERSORT (https://cibersort.stanford.edu/), xCell (https://xcell.ucsf.edu/), TIMER 2.0 (http://timer.cistrome.org/), and EPIC (http://epic.gfellerlab.org/). Wilcoxon’s rank-sum test was used to compare the immune cell infiltration proportion between primary and metastatic samples. Immune components with a *P* value of less than 0.05 in at least 2 datasets were retained. Pearson’s correlation analysis was used to estimate correlation between gene expression values and immune scores, with a *P* value of less than 0.05 considered statistically significant.

### Metabolism analysis.

Six metabolic pathways were collected from the Kyoto Encyclopedia of Genes and Genomes (KEGG, https://www.genome.jp/kegg/) database. Enrichment scores of these pathways in each sample were quantified using single-sample gene set enrichment analysis (ssGSEA). Additionally, metabolite protein interactions (MPIs) were collected from Chen et al. and the directed MPI sub-graph of interesting genes was extracted (40).

### scRNA-seq data processing and analysis.

The single-cell RNA expression profiles of 7 primary and 2 metastatic OS samples were acquired from GSE152048. The scRNA-seq data were processed with the R package “Seurat” (v 4.2.1). Cells with fewer than 300 genes or genes expressed in fewer than 3 cells were filtered out. Cells where more than 10% of transcripts derived from the mitochondria were also excluded. The data were normalized using “NormalizeData,” and the top 3000 highly variable genes were identified with “FindVariableFeatures.” Data from 9 samples were merged using the “IntegrateData.” The integrated dataset was scaled, and the top 30 principal components were used as inputs for graph-based cell clustering. Different resolutions were used in resolution steps of 0.05 for clustering, and the clustering hierarchy was investigated by plotting a clustree (41). Use of results of clustering at resolution 0.55 for TILs resulted in a total of 12 clusters; use of results of clustering at resolution 0.45 for myeloid cells resulted in a total of 13 clusters (Supplemental Figures 5 and 6). Clustering results were visualized using t-SNE with Seurat functions RunTSNE. Cell types were annotated based on the well-known cellular markers derived from the literature or the CellMarker database (42, 43). OBs were divided into *SNRNP70*^+^ OB (expression value > 0) and *SNRNP70*^–^ OB (expression value = 0) cells based on the expression of *SNRNP70*. The CellChat method was employed to analyze cell–cell interactions using the “CellChat” package (v1.4.0) (44).

### Cell culture and reagents.

The human OS cell lines U2OS and SAOS2 were cultured in McCOY’s 5A medium (MeilunBio). The human OS cell lines 143B and MG63 were cultured in minimal essential medium (MEM) (Sigma-Aldrich). The normal osteoblast cell line hFOB1.19 was cultured in Dulbecco’s modified Eagle’s medium (DMEM). All cells were supplemented with 10% FBS (Biological Industries) and 1% penicillin/streptomycin (Beyotime), and incubated at 37°C with 5% CO_2_. The OS cell lines (authenticated by single tandem repeat [STR] identification; see supplemental material) were purchased from Wuhan Procell. The normal osteoblast cell line (authenticated by STR identification; see supplemental material) was purchased from Shanghai Gaining Biological.

### siRNA and overexpression plasmid assay.

The full length of human SNRNP70 and CD55-Δe10 with a FLAG tag was amplified and subcloned into the pcDNA3.1 vector. An empty vector was used as a negative control (GeneCreate). Three siRNAs targeting CD55-Δe10 were synthesized for CD55-Δe10 knockdown, and a scrambled siRNA was synthesized for the negative control (RiboBio). The siRNAs and plasmids were then transfected using Lipofectamine 2000 (Invitrogen) according to the manufacturer’s instructions.

### Western blotting.

Cell lines or tumor tissues were harvested and lysed with RIPA buffer (Beyotime) supplemented with protease inhibitors. The protein sample (60 μg) was resolved in 10% SDS-PAGE and transferred to pure nitrocellulose (Pall Life Sciences), and then blocked with 5% fat-free dry milk for 1 hour. The membranes were incubated with primary antibodies against β-actin (1:2000, Proteintech, 20536-1-AP) and SNRNP70 (1:500, Abclonal, A14786) at 4°C overnight. Western blot (WB) bands were imaged using Odyssey (Gene Company Limited) and quantified with Image Studio v5.2 software.

### Quantitative real-time PCR.

Total RNA was isolated using TRIzol reagent (Invitrogen) according to the manufacturer’s protocol. cDNA reverse transcription kits (5 All-in- One RT Master Mix, TransGene Biotech) were used to reverse transcribe total RNA. The relative mRNA level was detected by SYBR Green on the ABI 7500 Fast Real-time PCR system (Applied Biosystems). β-Actin was used as a control for normalization. Relative expression was calculated by the 2^–ΔΔCt^ method.

### Agarose gel electrophoresis.

RNA samples were loaded on 2% (w/v) agarose gel and then separated using electrophoresis in Tris-acetate/EDTA running buffer at 100 V for 30 minutes. Gel images were visualized using the ChemiDoc MP Imaging System (Bio-Rad).

### CCK-8 assay.

Cell viability was assessed after transfection using a cell counting 8 (CCK-8) kit (Meilun Biotechnology) according to the manufacturer’s instructions. The data were independently replicated at least 6 times to ensure the reliability of the data.

### EdU assay.

EdU assay kit (RiboBio) was used to assess cell proliferation. Labeling medium containing 300 μM EdU was incubated with cells transfected with siRNA or plasmid in 24-well plates (5 × 10^4^ cells/well) for 2 hours. They were fixed with 4% paraformaldehyde for 30 minutes, followed by 10-minute permeabilization in 0.5% Triton X-100 and 30-minute reaction in 300 μL of 1× Apollo. Afterwards, Hoechst was then added to stain cell nuclei. EdU-positive cells were calculated under a fluorescence microscope (Leica).

### Wound-healing assay.

The OS cells were seeded into 6-well culture plates. Upon reaching 70% confluence, the cells were transfected with siRNA or plasmid. Subsequently, a scratch was gently created in the cell monolayer using a 200 μL pipette tip. The images were captured using a Nikon Ts100 microscope at both 0 and 24 hours after scratching. The wound area was quantified using ImageJ software (NIH). The migration rate (%) was calculated using the equation, migration rate = 1 – (24-hour scratch area/0-hour scratch area) × 100.

### Transwell assay.

The Transwell assay was performed using a 24-well Transwell chamber system (8-μm pore size, 6.5-mm diameter; Costar). In brief, 1 × 10^4^ cells transfected with siRNA or plasmid suspended in 200 μL of FBS-free medium were placed in the upper compartment, while 800 μL of medium containing 10% FBS was added to the lower compartment. Following a 48-hour incubation period, the cells were fixed and subsequently stained with crystal violet (Biosharp) to evaluate their invasive ability.

### Colony formation assay.

OS cells transfected with siRNA or plasmid were seeded in 6-well culture plates at a concentration of 500 cells per well. After 14 days of incubation, cells were fixed with methanol for 10 minutes and stained with 0.1% crystal violet for 15 minutes. Colonies were air-dried and counted. The experiments were repeated 3 times to ensure reliability of the results.

### RIP assay.

Cells were harvested and digested using RIP lysis buffer containing 10 mM KCl, 5 mM MgCl_2_, 10 mM HEPES (pH = 7.0), 0.5% NP-40, 1 mM DTT, 100 U/mL RRI, 20 μL/mL protease inhibitor, and 2 mM vanadyl ribonucleotide complex solution. Subsequently, the cell lysate was mixed with 3 μg of IP-grade anti-SNRNP70 antibody and incubated overnight at 4°C, while mouse IgG antibody served as a control. The reaction mixture was then supplemented with 40 μL of Protein A/G beads and allowed to react for 4 hours. The resulting precipitated RNA was extracted, purified, and analyzed by qRT-PCR and agarose gel electrophoresis.

### RNA pulldown.

In the RNA pulldown assay, streptavidin beads (Thermo Fisher Scientific, 11205D) were washed twice with 1 mL lysis buffer. Subsequently, the beads were blocked with 1 mL lysis buffer containing 5% BSA and 100 μg tRNA at 4°C for 1 hour. OS cells stably expressing FLAG-SNRNP70 were lysed in 1 mL lysis buffer and subjected to 3 rounds of sonication. The assay was then performed using an RNA pulldown kit (BersinBio) according to the manufacturer’s protocol. The coprecipitated protein was detected using WB analysis.

### Lentiviral infection.

The lentiviruses harboring siSNRNP70 and siNC were constructed by Genechem. OS 143B cells were infected with the lentivirus, and infected cells were subsequently selected using puromycin (1 μg/mL, Beyotime) for 7 days. The efficiency of knockdown was confirmed by WB and qRT-PCR analyses.

### Subcutaneous xenograft model.

Female BALB/c nude mice, approximately 4–6 weeks old, were purchased from Vital River and housed under pathogen-free conditions. 143B cells (5 × 10^6^ cells per mouse) infected with recombinant lentiviral vectors containing siSNRNP70 and siNC were subcutaneously injected into nude mice. Tumor size was measured every week, and tumor volume was calculated using the formula: *V* (mm^3^) = (*L* × *W*^2^)/2, where *L* represents the long axis and *W* represents the short axis of the tumor. Four weeks later, the mice were euthanized, and their tumors were isolated and weighed.

### Lung metastatic model.

To establish lung metastasis models, 143B cells (5 × 10^6^ cells per mouse) infected with recombinant lentiviral vectors containing siSNRNP70 were injected into the tail vein of nude mice. Four weeks later, metastatic tumors in the lungs were visualized using the IVIS system after D-luciferin injection. After IVIS measurement, the mice were euthanized, and their lungs were excised for further analysis.

### IHC assay.

The tumor tissues were fixed in 4% paraformaldehyde and embedded in paraffin. Tissue sections were then deparaffinized and rehydrated, followed by antigen retrieval through heat mediation in citrate buffer. The sections were treated with 3% H_2_O_2_ in methanol for 30 minutes and blocked with normal goat serum for 1 hour. Subsequently, the sections were incubated with anti–Ki-67 antibody (1:2000; Proteintech, 28074-1-AP) overnight at 4°C, washed with PBS, and then incubated with secondary antibodies for 30 minutes at room temperature. Next, the sections were stained with a DAB staining solution and counterstained with hematoxylin for 2 minutes. Images were captured using an Olympus IX73 microscope.

### H&E staining.

After routine deparaffination, the sections were washed with double-distilled H_2_O and stained with hematoxylin for 10 minutes. The color was differentiated with 1% hydrochloric acid alcohol for 3–5 seconds. Subsequently, the sections were dipped in distilled H_2_O for 30 seconds, followed by dipping in 95% alcohol, and then stained with eosin for 1 minute. Alcohol, dimethylbenzene, and neutral gum were used sequentially for dehydration, clearing, and sealing, respectively. Finally, the sections were observed under an Olympus IX73 microscope.

### Statistics.

All statistical analyses were performed with GraphPad Prism 9.0. Quantitative data are presented as the mean ± SEM. The significance of differences between 2 groups was determined using an unpaired, 2-tailed Student’s *t* test. For experiments with more than 2 independent groups, 1-way ANOVA was used to assess the statistical significance. A *P* value of less than 0.05 was considered statistically significant.

### Study approval.

The use of clinical cancer samples was approved by the Medical Ethics Committee of Henan Cancer Hospital (protocol number 2022-200-001) and each patient signed informed consent. All animal experiments were conducted in accordance with the guidelines of the American Association for the Accreditation of Laboratory Animal Care International and the NIH *Guide for the Care and Use of Laboratory Animals* (NIH publication no. 85-23, revised 1996). All procedures were approved by the Institutional Animal Care and Use Committee of Harbin Medical University (ethical approval number IRB2022724).

### Data availability.

The datasets analyzed during the current study are available from the public databases. Individual values for all other data are available in the Supporting Data Values file.

## Author contributions

BY and YG designed the project. WL and LW wrote the manuscript. LW and WT collected data and performed the bioinformatic analysis. WL, WJ, DB, YW, BX, and JF completed the experiments part. HL and PZ provided valuable suggestions for the manuscript. All authors read and approved the final manuscript.

## Supplementary Material

Supplemental data

Unedited blot and gel images

Supporting data values

## Version 1 12/20/2024

Electronic publication
