# Supplementary material for: SNRNP70 regulates the splicing of CD55 to promote osteosarcoma progression
Source: JCI Insight. 2024 Dec 20;9(24):e185269. doi: 10.1172/jci.insight.185269 (PMC11665567; doi:10.1172/jci.insight.185269)
Supplement: Supplemental data [file jciinsight-9-185269-s146.pdf]

Supplementary Tables

Supplementary Table 1. The osteosarcoma datasets used in this study.

Data	Adjacent samples	Osteosarcoma Samples		Data Source
		Metastatic	Primary	
GSE14359	/	8	10	http://www.ncbi.nlm.nih.gov/geo/
GSE14827	/	9	18	http://www.ncbi.nlm.nih.gov/geo/
GSE21257	/	34	19	http://www.ncbi.nlm.nih.gov/geo/
GSE32981	/	11	12	http://www.ncbi.nlm.nih.gov/geo/
GSE73166	/	7	3	http://www.ncbi.nlm.nih.gov/geo/
TARGET	/	22	65	https://ocg.cancer.gov/programs/target/
GSE42352	3	84		http://www.ncbi.nlm.nih.gov/geo/
SRP193919	4	16		https://www.ncbi.nlm.nih.gov/sra/
SRP090849	/	6	60	https://www.ncbi.nlm.nih.gov/sra/

Supplementary Table 2. The statistics of differentially expressed genes between primary and metastatic samples.

Data	Up-regulated genes	Down-regulated genes	Method
GSE14359	1507	1358	Student's t test
GSE14827	148	307	Student's t test
GSE21257	1395	952	Student's t test
GSE32981	275	359	Student's t test
GSE73166	975	673	Student's t test
TARGET	1612	1641	DESeq2
SRP090849	1763	3155	DESeq2

Supplementary Figures

Supplementary Figure 1. Consistently differentially expressed genes enriched in immune and metabolic related pathways.

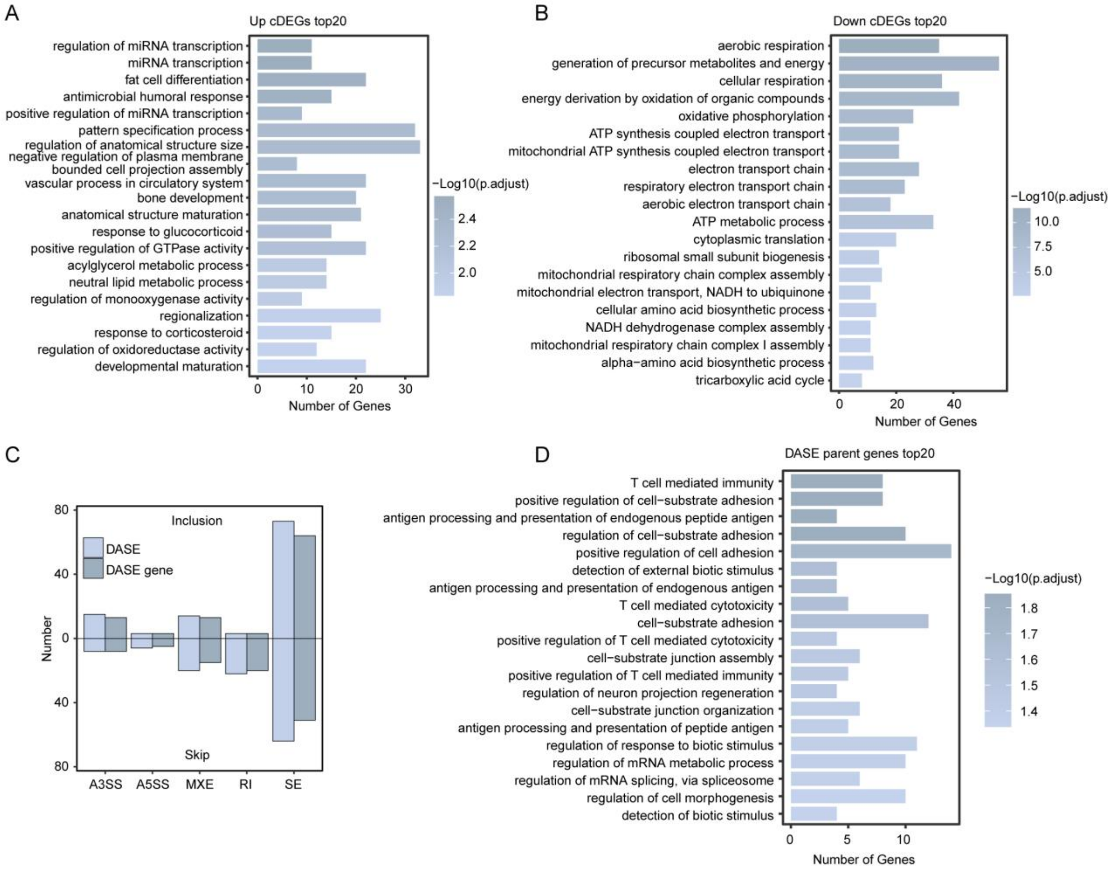

(A and B) GO pathway enrichment analyses of up-regulated consistently differentially expressed genes (cDEGs) (A), and down-regulated cDEGs (B). (C) Statistics of five splicing events. (D) GO pathway enrichment analysis of differentially alternatively spliced event (DASE) parent genes. Statistical significance was calculated using hypergeometric test (A, B and D).

Supplementary Figure 2. Revealing the regulatory role of the twenty specific genes in metastatic osteosarcoma.

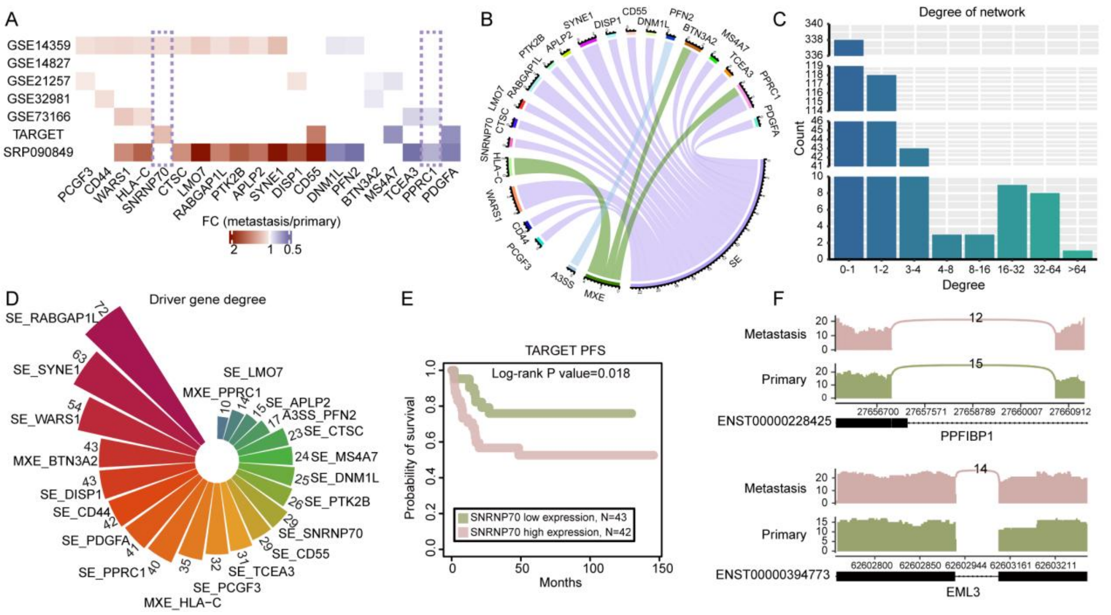

(A) Expression levels of the twenty specific genes in seven datasets. (B) Types of DASEs of the twenty specific genes. (C and D) Histograms showing the degree distribution of nodes for all genes (C) and the twenty specific genes (D). (E) Kaplan–Meier analysis illustrating the impact of *SNRNP70* expression on the progression free survival in TARGET. (F) Sashimi plot depicting splicing changes of *PPFIBP1* and *EML3* in metastatic osteosarcoma (OS). Statistical significance was calculated using two-tiled Wilcoxon rank-sum test (A) and log-rank test (E).

Supplementary Figure 3. SNRNP70 shows correlation with metabolic pathways.

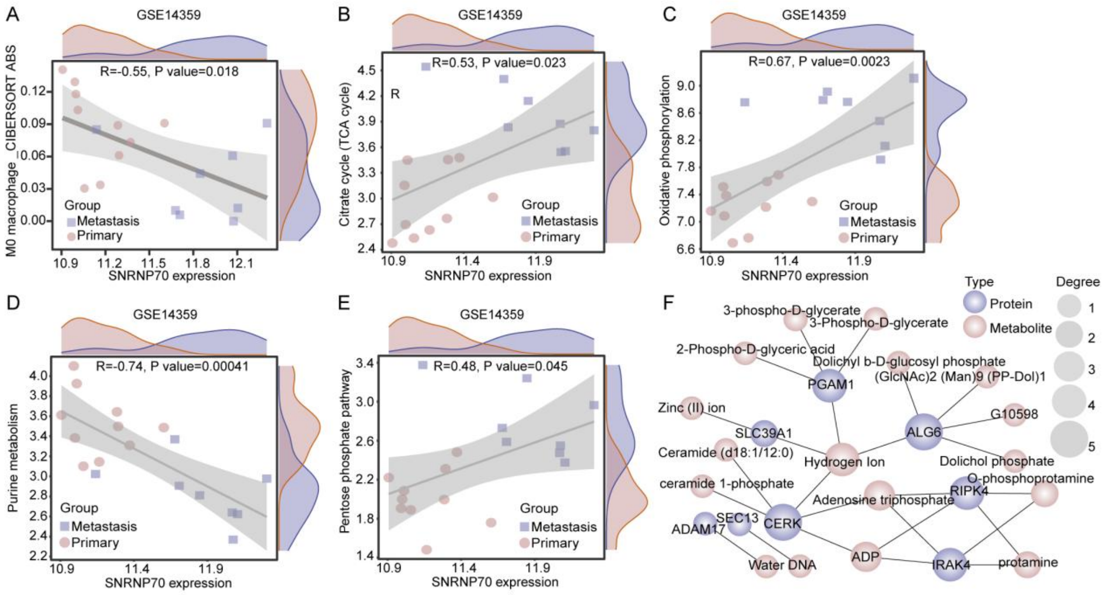

(A) Correlation analysis between *SNRNP70* expression and infiltration proportions of M0 macrophages in GSE14359 (Pearson correlation analysis). (B-E) Correlation analysis between *SNRNP70* expression and activity of metabolic pathway in GSE14359 (Pearson correlation analysis). (F) The subgraph of cDEGs regulated by *SNRNP70* is extracted from metabolite protein interactions (MPIs).

Supplementary Figure 4. Analysis of scRNA-seq dataset of OS.

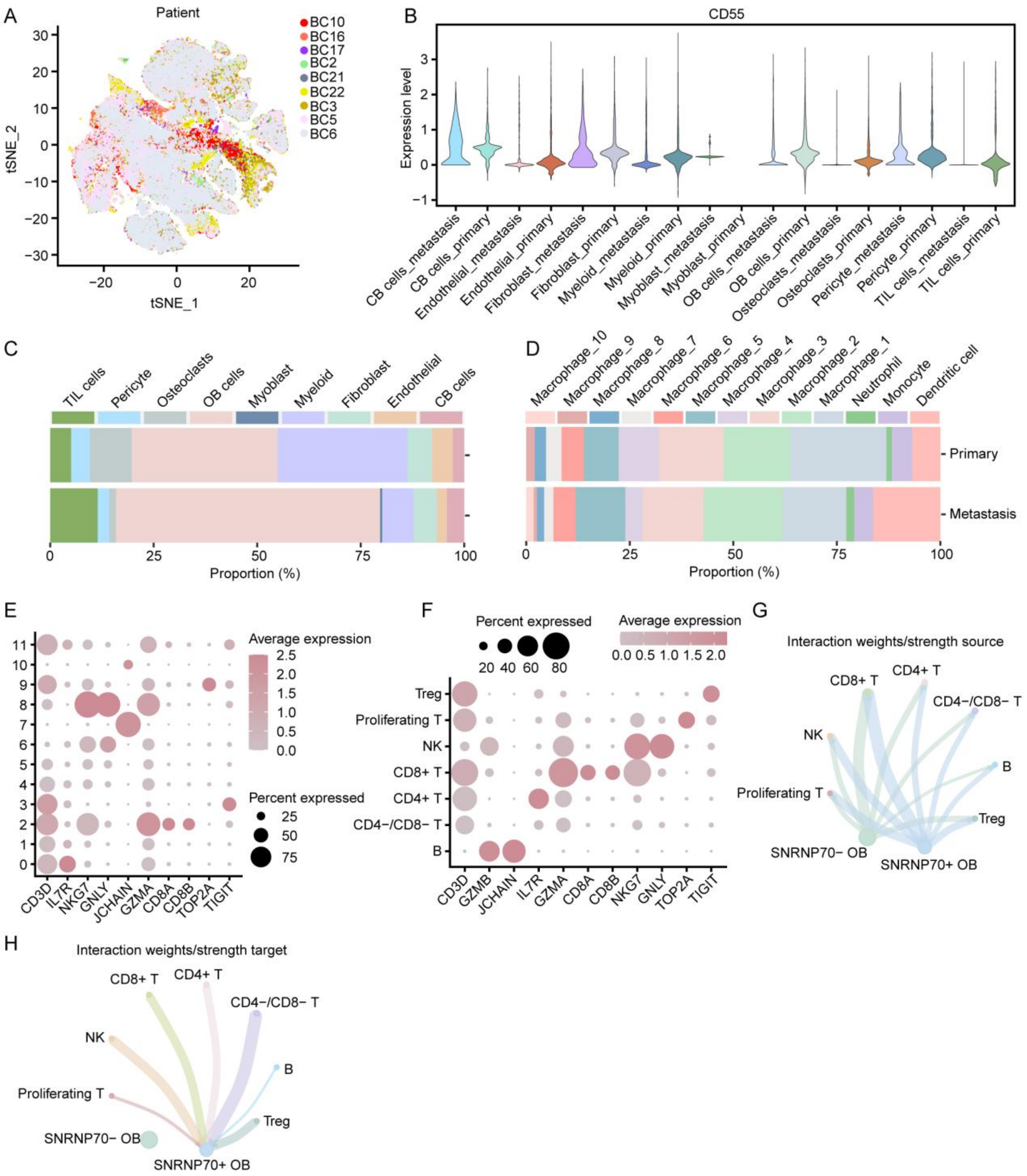

(A) t-SNE visualization of 91,430 cells analyzed by scRNA-seq and integrated across 9 OS samples, color-coded by sample. (B) Violin plot showing *CD55* expressions across the 9 clusters in primary and metastatic OS. (C and D) The proportion of 9 clusters (C) and myeloid cell subsets (D) in primary versus metastatic tissues. (E and F) Dot plots showing the signature gene expressions in tumor-infiltrating lymphocytes (TILs). (G and H) Circle plot of interaction weights between myeloid cells and *SNRNP70*$^{+}$ OB cells or *SNRNP70*$^{-}$ OB cells.

Supplementary Figure 5. The clustering tree of TILs at multiple resolutions.

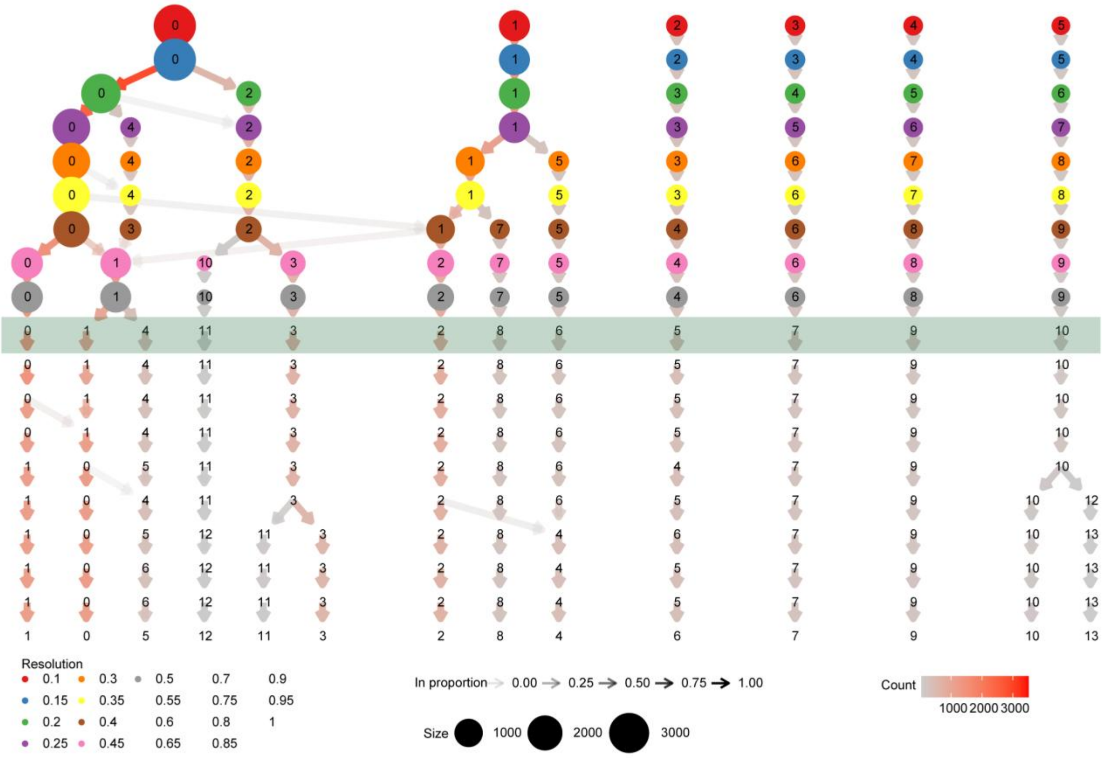

The size of node denotes the number of cells in a cluster. The color of node represents the resolution value. Edges are color-coded to reflect the quantity of cells they correspond to, with transparency indicating the proportion of the incoming node.

Supplementary Figure 6. The clustering tree of myeloid cells at multiple resolutions.

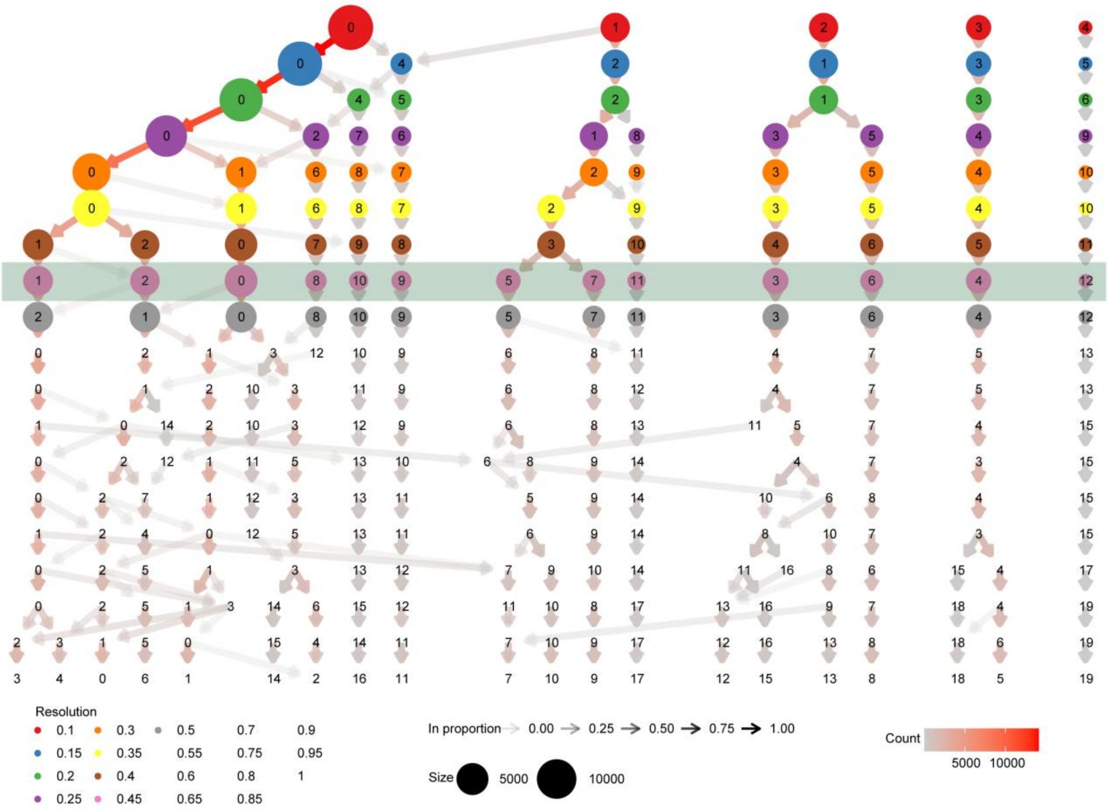

The size of node denotes the number of cells in a cluster. The color of node represents the resolution value. Edges are color-coded to reflect the quantity of cells they correspond to, with transparency indicating the proportion of the incoming node.

细胞遗传质量鉴定检测

Cell Line Authentication Service

STR 基因型检测报告

送检单位：上海酶研生物科技有限公司

检品名称：细胞系

委托单位：上海翼和应用生物技术有限公司

报告日期：2018-06-01

上海翼和应用生物技术有限公司 联系地址：上海市漕河泾开发区桂平路 471 号桂果园 8 号楼 4F 室
网址：http://www.biowing.com.cn/ 邮箱：biowing@vip.163.com 服务热线：021-33559491

报 告 说 明

1. 本报告只对送检的来样负责。
2. 检验报告上的检验结果和检验单位名称，未经同意不得用于广告、评优及商业宣传。
3. 对本报告有异议，请于收到报告之日起十五日内以书面方式提出，逾期不予受理。
4. 对纸质检验报告涂改、增删，或未加盖检验单位印章的复印件均无效。

上海翼和应用生物技术有限公司 联系地址：上海市漕河泾开发区桂平路 471 号桂果园 8 号楼 4F 室
网址：http://www.biowing.com.cn/ 邮箱：biowing@vip.163.com 服务热线：021-33559491

样品信息

样品编号：

客户样本编号	公司编号
143B	20180528-01

样品数量：1

样品性状：细胞系

检测项目：STR

送检单位：酶研生物

检测方法：用 Axygen 的基因组抽提试剂盒提取 DNA，采用 20- STR 扩增方案扩增，在 ABI 3730XL 型遗传分析仪上对 STR 位点和性别基因 Amelogenin 进行检测。

上海翼和应用生物技术有限公司 联系地址：上海市漕河泾开发区桂平路 471 号桂果园 8 号楼 4F 室
网址：http://www.biowing.com.cn/ 邮箱：biowing@vip.163.com 服务热线：021-33559491

检 测 结 果

(一)　检验基本情况

	多等位基因	匹配细胞系	细胞库	EV 值	匹配说明
20180528-01	无	143B	DSMZ	1.00	完全匹配

样本基因型检验结果

- 多等位基因指三等位及以上基因现象。
- 本次检测各细胞分型结果良好。

(二)　各样本描述

- 20180528-01：该株细胞 DNA 分型在细胞系检索中找到**完全匹配**的细胞系，DSMZ 数据库显示细胞名为 **143B** ，细胞号对应 **CRL-8303** 。本次检测在该细胞系中**没有发现多等位基因**。

  备注：待测细胞系与收录于 ATCC, DSMZ, JCRB 和 RIKEN 数据库的细胞系 STR 数据进行比对，未收录于以上细胞库的细胞系将无法匹配。

上海翼和应用生物技术有限公司　联系地址：上海市漕河泾开发区桂平路 471 号桂果园 8 号楼 4F 室
网址：http://www.biowing.com.cn/　邮箱：biowing@vip.163.com　服务热线：021-33559491

（三）样本分型结果

细胞 20180528-01 的 STR 位点和 Amelogenin 位点的基因分型结果						
Loci	送检细胞 STR 信息			细胞库细胞 STR 信息		
	送检细胞名：143B			细胞库细胞名：143.98.2		
	Allele1	Allele2	Allele3	Allele1	Allele2	Allele3
D5S818	13	13		13	13	
D13S317	12	12		12	12	
D7S820	11	12		11	12	
D16S539	10	13		10	13	
VWA	18	18		18	18	
TH01	6	6		6	6	
AMEL	X	X		X	X	
TPOX	11	11		11	11	
CSF1PO	12	12		12	12	
D12S391	20	21				
FGA	24	24				
D2S1338	24	25				
D21S11	31.2	32.2				
D18S51	17	17				
D8S1179	11	14				
D3S1358	15	15				
D6S1043	18	18				
PENTAE	7	12				
D19S433	13	13				
PENTAD	9	10				

上海翼和应用生物技术有限公司 联系地址：上海市漕河泾开发区桂平路 471 号桂果园 8 号楼 4F 室
网址：http://www.biowing.com.cn/ 邮箱：biowing@vip.163.com 服务热线：021-33559491

其他说明

(一) 分型方案及位点分布

	方案 1	方案 2	方案 3	方案 4
1	TH01	TPOX	D3S1358	AMEL
2	D12S391	VWA	D13S317	D5S818
3	D7S820	D8S1179	D6S1043	D2S1338
4	CSF1PO	PENTAD	D16S539	D21S11
5	FGA		D19S433	D18S51
6	PENTAE			

实验方案及位点

(二) STR 数据库比对

本公司采用 DSMZ tools 进行细胞系比对，其中包含来自于 ATCC, DSMZ, JCRB 和 RIKEN 数据库的 2455 个细胞系 STR 数据。如果待检测细胞未收录于以上细胞库或这是自行建立的新细胞系将无法进行比对，用户需根据细胞分型结果自行与其他数据库进行比对。

(三) 文献引用参考

1．Authentication testing of HEK 293T and HeLa cell lines have been performed by Shanghai Biowing Applied Biotechnology Co.,Ltd via STR profiling. STR profiles match the standards re commended for HEK 293T and HeLa cell lines authentication

2．AGS, NCI-N87, HGC-27 and HEK293 were STR-authenticated on Dec. 8, 2015 by Shanghai Biowing Applied Biotechnology Co. LTD, Shanghai, China

主要实验人员：张佳男

复核人: 钱宁

负责人：白杨

签发日期：2018 年 06 月 01 日

上海翼和应用生物技术有限公司 联系地址：上海市漕河泾开发区桂平路 471 号桂果园 8 号楼 4F 室
网址：http://www.biowing.com.cn/ 邮箱：biowing@vip.163.com 服务热线：021-33559491

细胞遗传质量鉴定检测

Cell Line Authentication Service

STR 基因型检测报告

送检单位：上海盖宁生物科技有限公司
检品名称：细胞系
委托单位：上海翼和应用生物技术有限公司
报告日期：2018-02-07

上海翼和应用生物技术有限公司 联系地址：上海市漕河泾开发区桂平路 471 号桂果园 8 号楼 4F 室
网址：http://www.biowing.com.cn/ 邮箱：biowing@vip.163.com 服务热线：021-33559491

样 品 信 息

样品编号：

客户样本编号	公司编号
HFOB.1.19	20180118-01

样品数量： 1

样品性状： 细胞系

检测项目： STR

送检单位： 盖宁生物

检测方法： 用 Axygen 的基因组抽提试剂盒提取 DNA，采用 20- STR 扩增方案扩增，在 ABI 3730XL 型遗传分析仪上对 STR 位点和性别基因 Amelogenin 进行检测。

上海翼和应用生物技术有限公司 联系地址：上海市漕河泾开发区桂平路 471 号桂果园 8 号楼 4F 室
网址：http://www.biowing.com.cn/ 邮箱：biowing@vip.163.com 服务热线：021-33559491

检 测 结 果

(一)　检验基本情况

	多等位基因	匹配细胞系	细胞库	EV 值	匹配说明
20180118-01	无	hFOB 1.19	DSMZ	0.94	基本匹配

样本基因型检验结果

- 多等位基因指三等位及以上基因现象。
- 本次检测各细胞分型结果良好。

(二)　各样本描述

- 20180118-01：该株细胞 DNA 分型在细胞系检索中找到**基本匹配**的细胞系，DSMZ 数据库显示细胞名为 **HFOB 1.19**，细胞号对应 **CRL-11372**。本次检测在该细胞系中**没有发现多等位基因**。

  备注：待测细胞系与收录于 ATCC, DSMZ, JCRB 和 RIKEN 数据库的细胞系 STR 数据进行比对，未收录于以上细胞库的细胞系将无法匹配。

（三）样本分型结果

细胞 20180118-01 的 STR 位点和 Amelogenin 位点的基因分型结果						
Loci	送检细胞 STR 信息			细胞库细胞 STR 信息		
	送检细胞名：HFOB.1.19			细胞库细胞名：hFOB 1.19		
	Allele1	Allele2	Allele3	Allele1	Allele2	Allele3
D5S818	11	11		11	12	
D13S317	11	12		11	12	
D7S820	8	10		8	10	
D16S539	9	13		9	13	
VWA	16	18		16	18	
TH01	7	9.3		7	9.3	
AMEL	X	X		X	X	
TPOX	11	11		11	11	
CSF1PO	10	13		10	13	
D12S391	20	23				
FGA	19	22				
D2S1338	23	24				
D21S11	29	32.2				
D18S51	10	17				
D8S1179	10	14				
D3S1358	17	18				
D6S1043	13	16				
PENTAE	8	11				
D19S433	13	15				
PENTAD	9	13				

其 他 说 明

(一) 分型方案及位点分布

	方案 1	方案 2	方案 3	方案 4
1	TH01	TPOX	D3S1358	AMEL
2	D12S391	VWA	D13S317	D5S818
3	D7S820	D8S1179	D6S1043	D2S1338
4	CSF1PO	PENTAD	D16S539	D21S11
5	FGA		D19S433	D18S51
6	PENTAE			

实验方案及位点

(二) STR 数据库比对

本公司采用 DSMZ tools 进行细胞系比对，其中包含来自于 ATCC, DSMZ, JCRB 和 RIKEN 数据库的 2455 个细胞系 STR 数据。如果待检测细胞未收录于以上细胞库或这是自行建立的新细胞系将无法进行比对，用户需根据细胞分型结果自行与其他数据库进行比对。

(三) 文献引用参考

1．Authentication testing of HEK 293T and HeLa cell lines have been performed by Shanghai Biowing Applied Biotechnology Co.,Ltd via STR profiling. STR profiles match the standards recommended for HEK 293T and HeLa cell lines authentication

2．AGS, NCI-N87, HGC-27 and HEK293 were STR-authenticated on Dec. 8, 2015 by Shanghai Biowing Applied Biotechnology Co. LTD, Shanghai, China

主要实验人员：张佳男

复核人: 龚礼瑾

负责人： 陈轶群

签发日期：2018 年 02 月 07 日

MG-63细胞STR鉴定报告

一、 材料处理和检验方法

取适量MG-63细胞(编号PC-H2023062814,1×10^6)使用TIANamp Genomic DNA Kit提取DNA，采用Microreader™21 ID System扩增20个STR位点和性别鉴定位点，使用智阅基因分析仪GenReader 7010进行PCR产物检测，使用GeneMapper Software6软件（Applied Biosystems）对检测结果进行分析，并与ExPASy数据库进行比对。

二、 检测结果

实验中阴性及阳性对照结果均正确。

MG-63细胞株的STR位点和Amelogenin位点的基因分型结果见附表，分型图谱见附图。

三、 分析说明

MG-63细胞株基因组DNA扩增后图谱清晰，分型结果良好。

四、 检验结论

1. MG-63细胞株DNA进行细胞STR分型结果显示，细胞株中未发现人类细胞交叉污染。
2. 该细胞株DNA分型在细胞库中找到与其细胞分型98.18%相匹配的细胞株，细胞株名称为MG-63。

附表1：细胞株MG-63的STR位点和Amelogenin位点的基因分型结果。

STR Loci	样品名称：PC-H2023062814	数据库名称：MG-63
Amelogenin	X, Y	X,Y
CSF1PO	10,12	10,12
D2S1338	17,24	17,24
D3S1358	15	15
D5S818	11,12	11,12
D7S820	10	10
D8S1179	13	13
D13S317	11	11
D16S539	11,12	11,12
D18S51	12,16	16
D19S433	13,14	13,14
D21S11	30	30
FGA	21,25	21,25
PentaD	9,13	9,13
PentaE	11,12	11,12
TH01	9.3	9.3
TPOX	8,11	8,11
vWA	16,19	16,19
D6S1043	12,17	
D12S391	15,20	
D2S441	10,11	
ExPASy数据库匹配度98.18%，匹配位点数17（https://web.expasy.org/cellosaurus-str-search/）		

备注：

1. 根据国际细胞鉴定委员会(ICLAC)制定的细胞 STR 鉴定标准，细胞系的匹配度≥80% 时，认为它们具有相关性，即衍生于共同的祖先细胞；匹配度在 55% 至 80% 之间，需要进一步验证相关性；小于 55%，表明两者不具有相关性。
2. 图谱有效峰为真实的 PCR 条带;小峰和非特异性条带在计算中忽略不计。
3. STR 数据比对结果默认 ExPASy，数据来源包括 ATCC，DSMZ，JCRB 等细胞库以及文献和资料记载，数据库入口 https://web.expasy.org/cellosaurus-str-search/。

网站：www.procell.com.cn
电话：400-999-2100；027-87287608
邮箱：sales@procell.com.cn
地址：湖北省武汉市高新大道858号生物医药产业园三期C4栋

附图1：MG-63细胞(编号PC-H2023062814)STR位点和Amelogenin位点的基因分型结果。

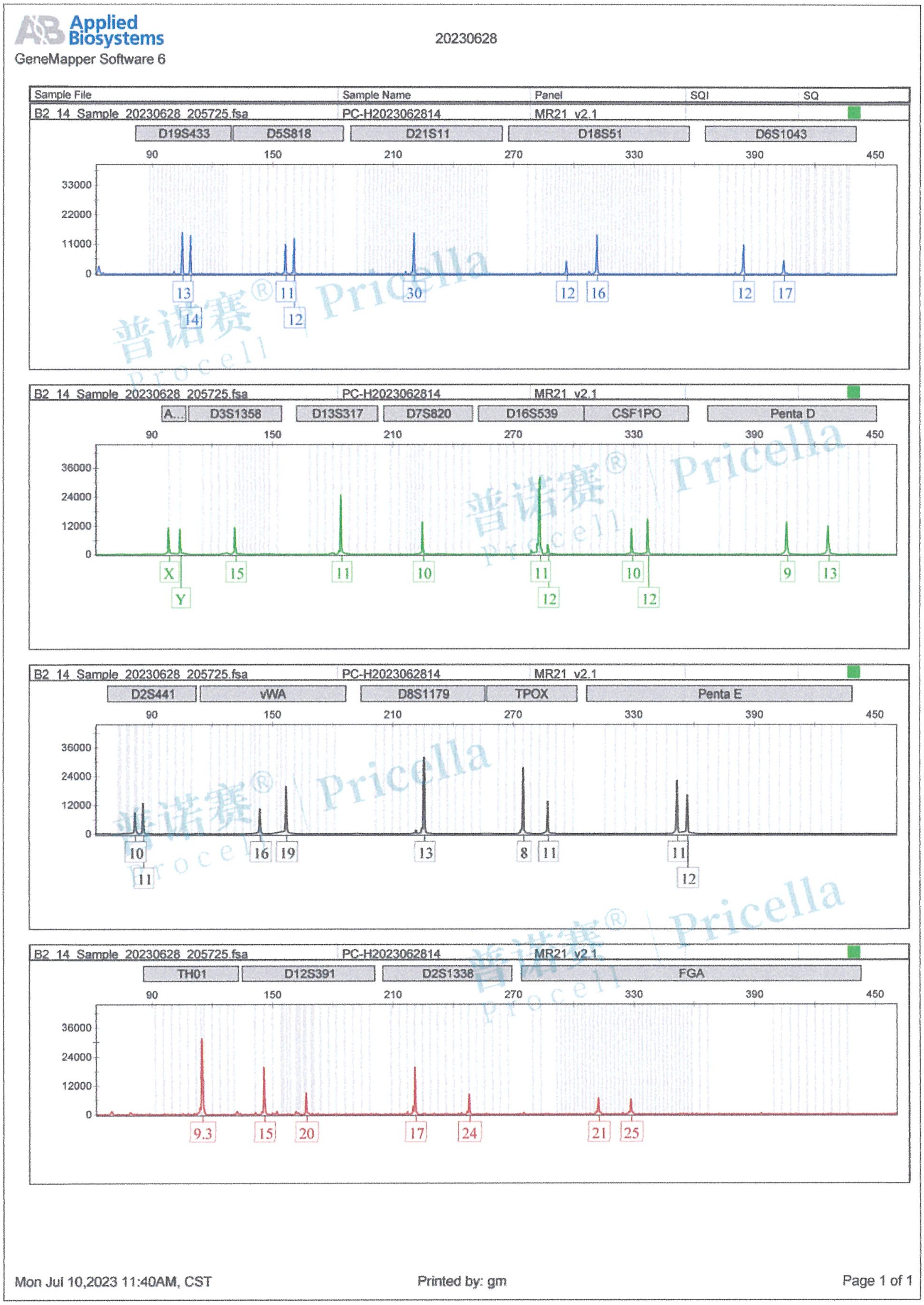

网站：www.procell.com.cn
电话：400-999-2100； 027-87287608
邮箱：sales@procell.com.cn
地址：湖北省武汉市高新大道858号生物医药产业园三期C4栋

Saos-2细胞STR鉴定报告

一、 材料处理和检验方法

取适量Saos-2细胞(编号PC-H2023012925,1×10^6)使用Microread Genomic DNA Kit提取DNA，采用Microreader™21 ID System扩增20个STR位点和性别鉴定位点，使用智阅基因分析仪GenReader 7010进行PCR产物检测，使用GeneMapper Software6软件（Applied Biosystems）对检测结果进行分析，并与ExPASy数据库进行比对。

二、 检测结果

实验中阴性及阳性对照结果均正确。

Saos-2细胞株的STR位点和Amelogenin位点的基因分型结果见附表，分型图谱见附图。

三、 分析说明

Saos-2细胞株基因组DNA扩增后图谱清晰，分型结果良好。

四、 检验结论

1. Saos-2细胞株DNA进行细胞STR分型结果显示，细胞株中未发现人类细胞交叉污染。
2. 该细胞株DNA分型在细胞库中找到与其细胞分型98.11%相匹配的细胞株，细胞株名称为Saos-2。

附表1：细胞株Saos-2的STR位点和Amelogenin位点的基因分型结果。

STR Loci	样品名称：PC-H2023012925	数据库名称：Saos-2
Amelogenin	X	X
CSF1PO	10	10
D2S1338	18	18
D3S1358	18	14,18
D5S818	12	12
D7S820	8,10	8,10
D8S1179	10,12	10,12
D13S317	12,13	12,13
D16S539	12,13	12,13
D18S51	15	15
D19S433	13	13
D21S11	28,30	28,30
FGA	22,25	22,25
PentaD	11,12	11,12
PentaE	14,19	14,19
TH01	6,9	6,9
TPOX	8	8
vWA	18	18
D6S1043	11,14	
D12S391	20	
D2S441	10,14	
ExPASy数据库匹配度98.11%，匹配位点数17（https://web.expasy.org/cellosaurus-str-search/）		

备注：

1. 根据国际细胞鉴定委员会(ICLAC)制定的细胞 STR 鉴定标准，细胞系的匹配度≥80% 时，认为它们具有相关性，即衍生于共同的祖先细胞；匹配度在 55% 至 80% 之间，需要进一步验证相关性；小于 55%，表明两者不具有相关性。
2. 图谱有效峰为真实的 PCR 条带;小峰和非特异性条带在计算中忽略不计。
3. STR 数据比对结果默认 ExPASy，数据来源包括 ATCC，DSMZ，JCRB 等细胞库以及文献和资料记载，数据库入口 https://web.expasy.org/cellosaurus-str-search/。

网站：www.procell.com.cn
电话：400-999-2100；027-87287608
邮箱：sales@procell.com.cn
地址：湖北省武汉市高新大道858号生物医药产业园三期C4栋

附图1：Saos-2细胞(编号PC-H2023012925)STR位点和Amelogenin位点的基因分型结果。

Applied Biosystems
GeneMapper Software 6

20230202_1

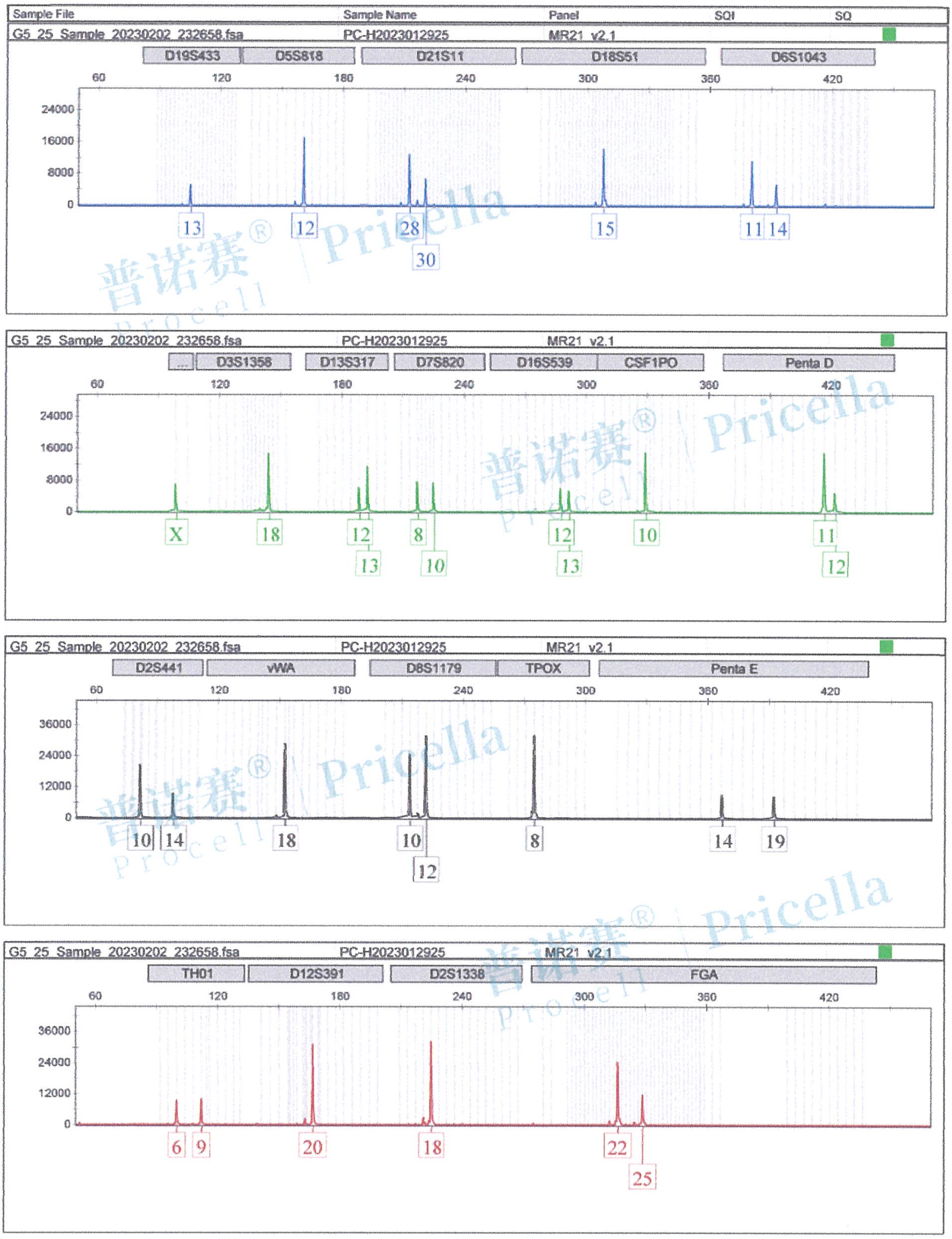

Mon Feb 06,2023 11:43AM, CST   Printed by: gm   Page 1 of 1

网站：www.procell.com.cn
电话：400-999-2100；027-87287608
邮箱：sales@procell.com.cn
地址：湖北省武汉市高新大道858号生物医药产业园三期C4栋

U-2 OS [U2OS]细胞STR鉴定报告

一、 材料处理和检验方法

取适量**U-2 OS [U2OS]**细胞(编号PC-H2022070106,1×10^{6})使用Microread Genomic DNA Kit提取DNA，采用Microreader™21 ID System扩增20个STR位点和性别鉴定位点，使用ABI 3730xl型遗传分析仪进行PCR产物检测，使用GeneMapper Software6软件（Applied Biosystems）对检测结果进行分析，并与ATCC、DSMZ、JCRB、ExPASy等数据库进行比对。

二、 检测结果

实验中阴性及阳性对照结果均正确。

U-2 OS [U2OS]细胞株的 STR 位点和 Amelogenin 位点的基因分型结果见附表，分型图谱见附图。

三、 分析说明

U-2 OS [U2OS]细胞株基因组DNA扩增后图谱清晰，分型结果良好。

四、 检验结论

1. **U-2 OS [U2OS]**细胞株 DNA 进行细胞 STR 分型结果显示，细胞株中未发现人类细胞交叉污染。
2. 该细胞株 DNA 分型在细胞库中找到与其细胞分型 98.36%相匹配的细胞株，细胞株名称为**U-2 OS [U2OS]**。

附表1：细胞株U-2 OS [U2OS]的STR位点和Amelogenin位点的基因分型结果

STR Loci	样品名称：PC-H2022070106	数据库名称：U-2 OS [U2OS]
Amelogenin	X	X
CSF1PO	13	13
D2S1338	20,24	20,24
D3S1358	16	16
D5S818	11	11
D7S820	11,12	11,12
D8S1179	12,14	12,14
D13S317	13	13
D16S539	11,12	11,12
D18S51	14	14
D19S433	15	15
D21S11	31	31
FGA	20,21	20
PentaD	9	9
PentaE	10,13	10,13
TH01	6,9.3	6,9.3
TPOX	11,12	11,12
vWA	14,18	14,18
D6S1043	11	11
D12S391	19,20	19,20
D2S441	10,14	10,14
ExPASy数据库匹配度98.36%，匹配位点数20（https://web.expasy.org/cellosaurus-str-search/）		

备注：

1. 根据国际细胞鉴定委员会(ICLAC)制定的细胞 STR 鉴定标准，细胞系的匹配度≥80% 时，认为它们具有相关性，即衍生于共同的祖先细胞；匹配度在 55% 至 80% 之间，需要进一步验证相关性；小于 55%，表明两者不具有相关性。
2. 图谱有效峰为真实的 PCR 条带;小峰和非特异性条带在计算中忽略不计。
3. STR 数据比对结果默认 ExPASy，数据来源包括 ATCC，DSMZ，JCRB 等细胞库以及文献和资料记载，数据库入口 https://web.expasy.org/cellosaurus-str-search/。

附图1：U-2 OS [U2OS]细胞STR位点和Amelogenin位点的基因分型结果

Applied Biosystems
GeneMapper Software 6

20220701

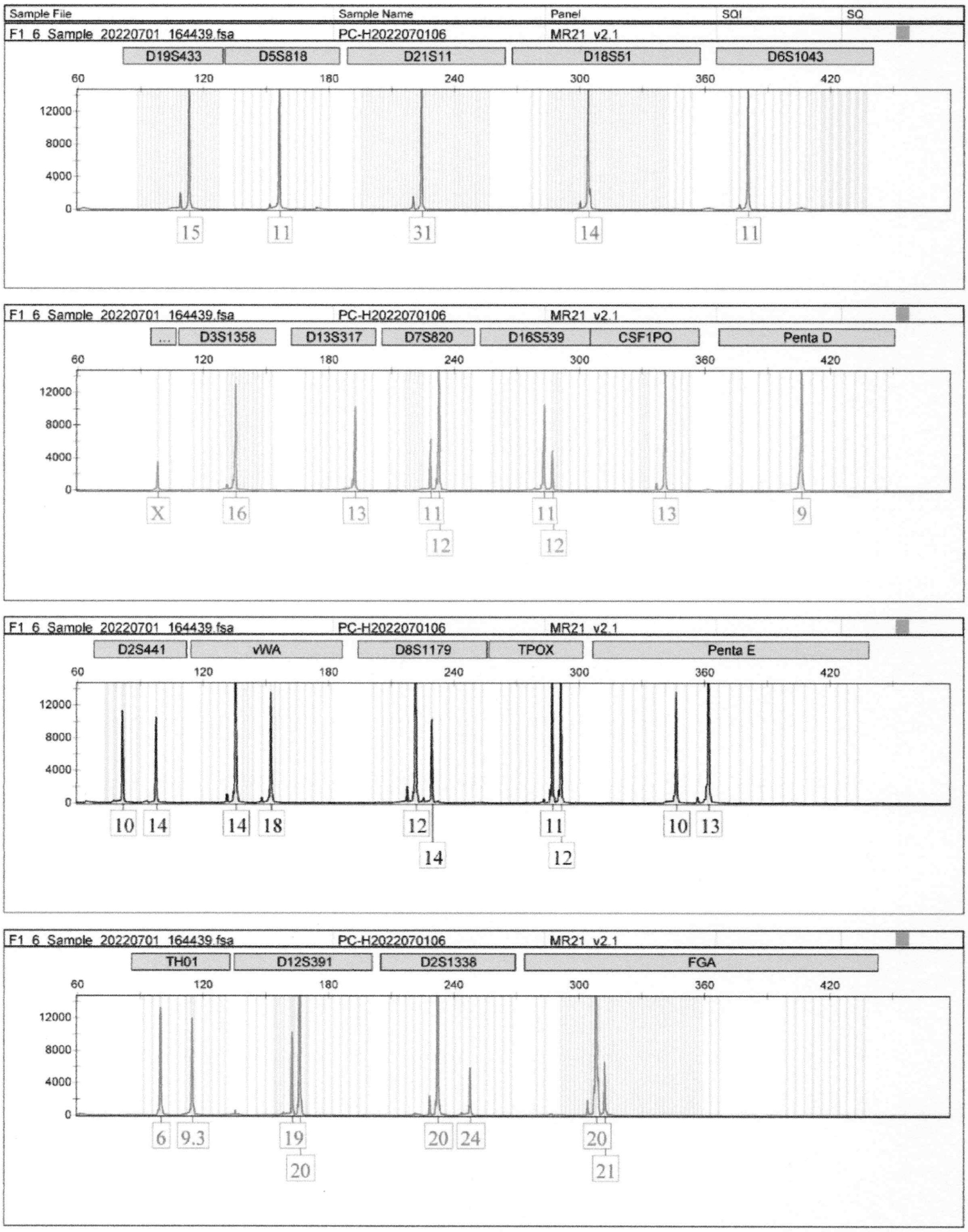

Mon Jul 18,2022 02:10PM, CST

Printed by: gm

Page 1 of 1
