# Supplementary material for: SNRNP70 regulates the splicing of CD55 to promote osteosarcoma progression
Source: JCI Insight. 2024 Dec 20;9(24):e185269. doi: 10.1172/jci.insight.185269 (PMC11665567; doi:10.1172/jci.insight.185269)

Fig.2F

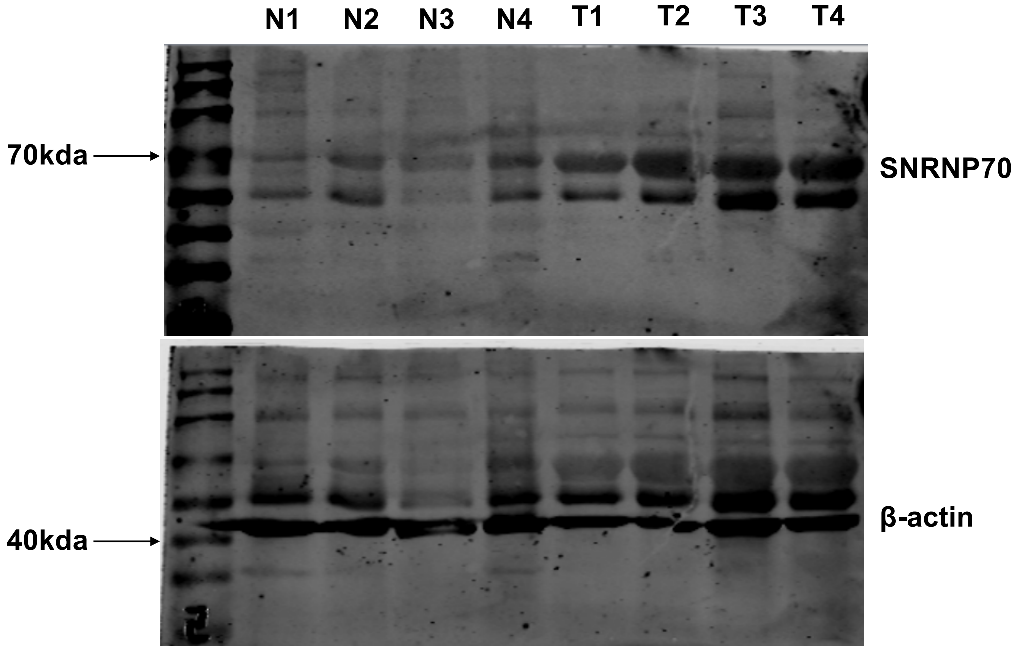

Fig.3G

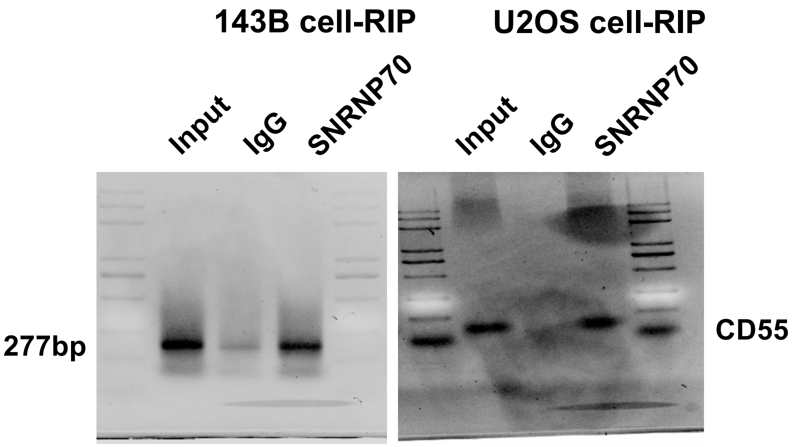
143B cell-RIP
U2OS cell-RIP
Input
IgG
SNRNP70
Input
IgG
SNRNP70
277bp
CD55

Fig.3H

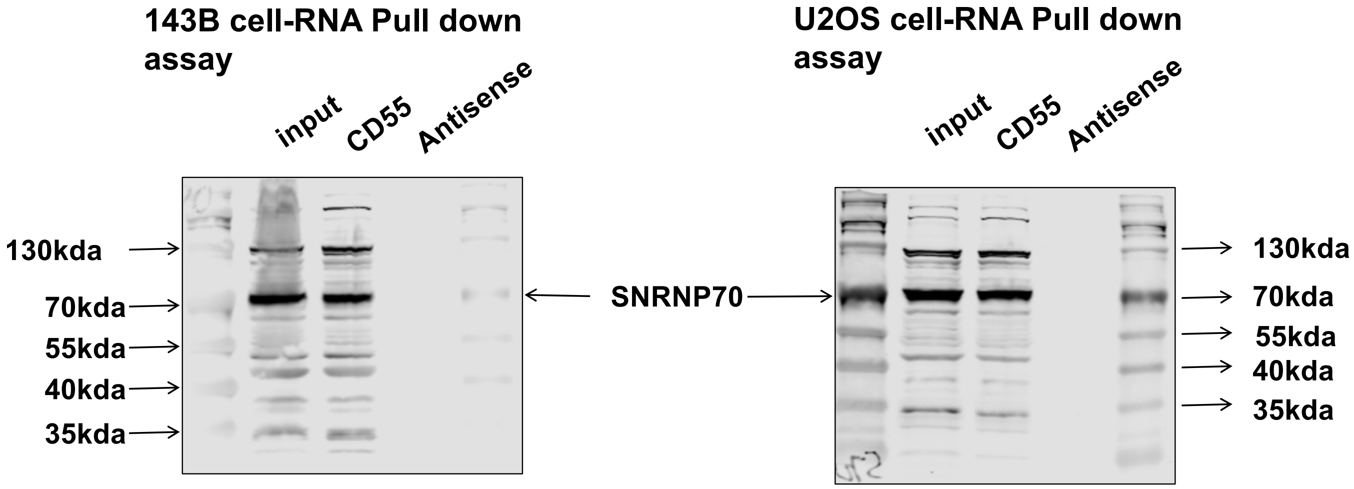
143B cell-RNA Pull down assay
input
CD55
Antisense
130kda
70kda
55kda
40kda
35kda
SNRNP70
U2OS cell-RNA Pull down assay
input
CD55
Antisense
130kda
70kda
55kda
40kda
35kda

Fig.3K

143B cell-SNRNP70 regulated the alternative splicing of CD55

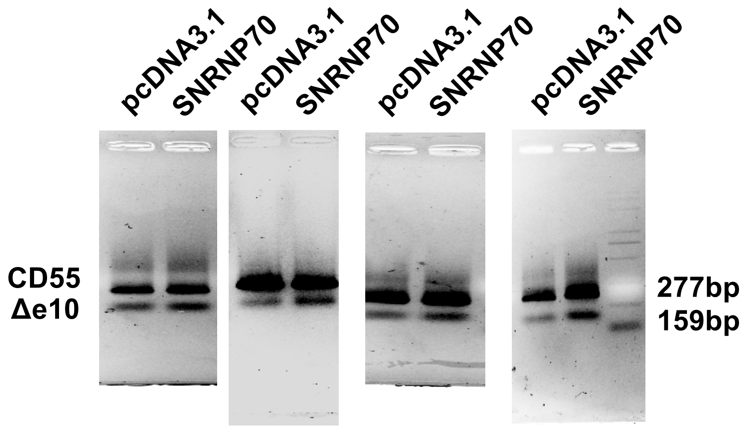

Fig.3K

U2OS cell-SNRNP70 regulated the alternative splicing of CD55

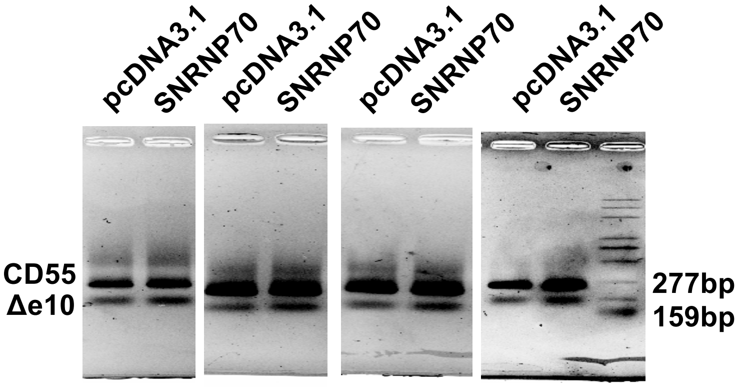

Fig.3L

Alternative splicing of CD55 in OS and normal bone cell lines

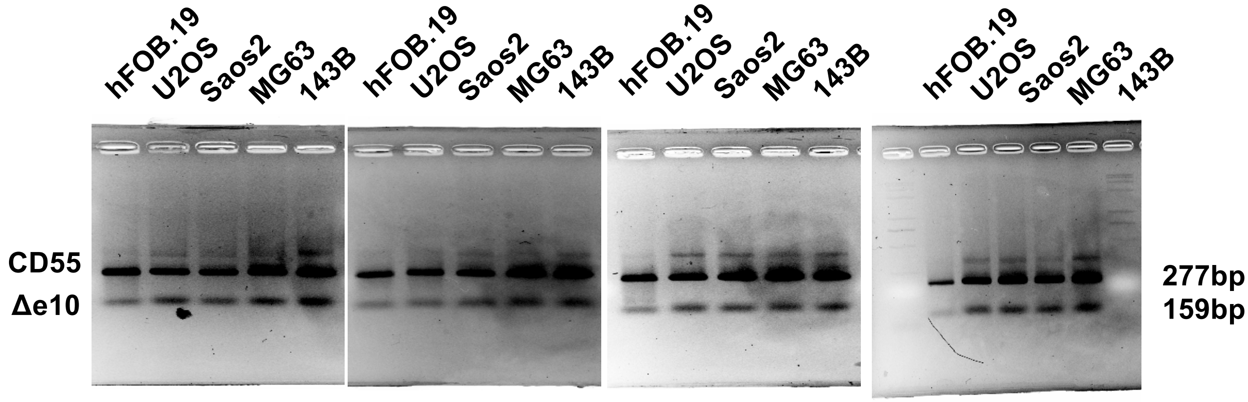

Fig.3I

143B cell-SNRNP70 overexpression(protein level)

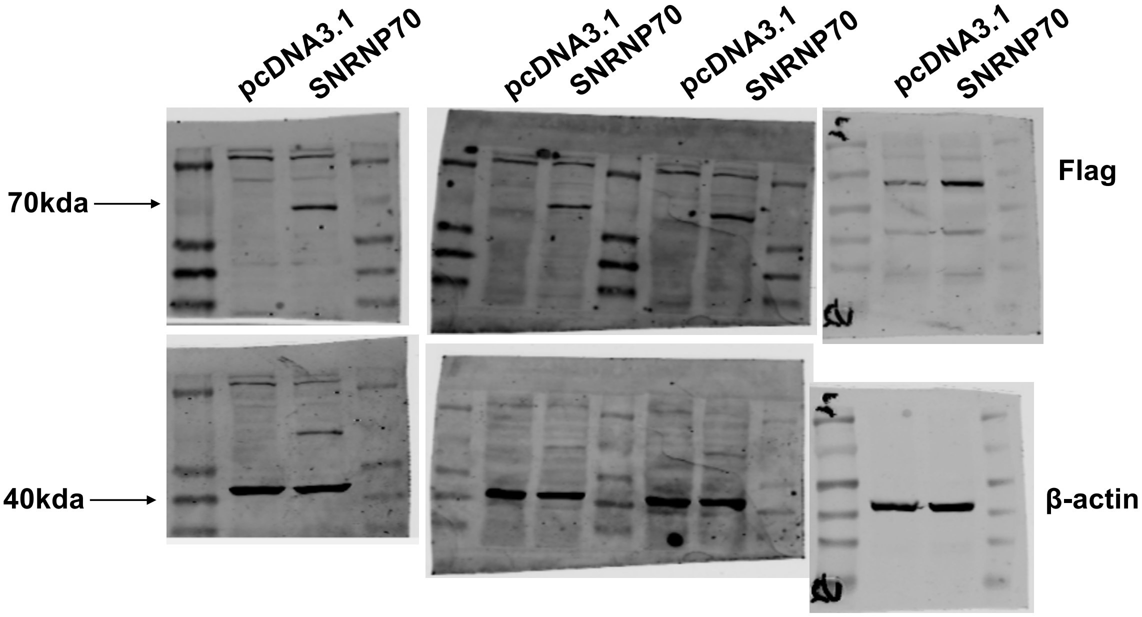

Fig.3I

U2OS cell-SNRNP70 overexpression(protein level)

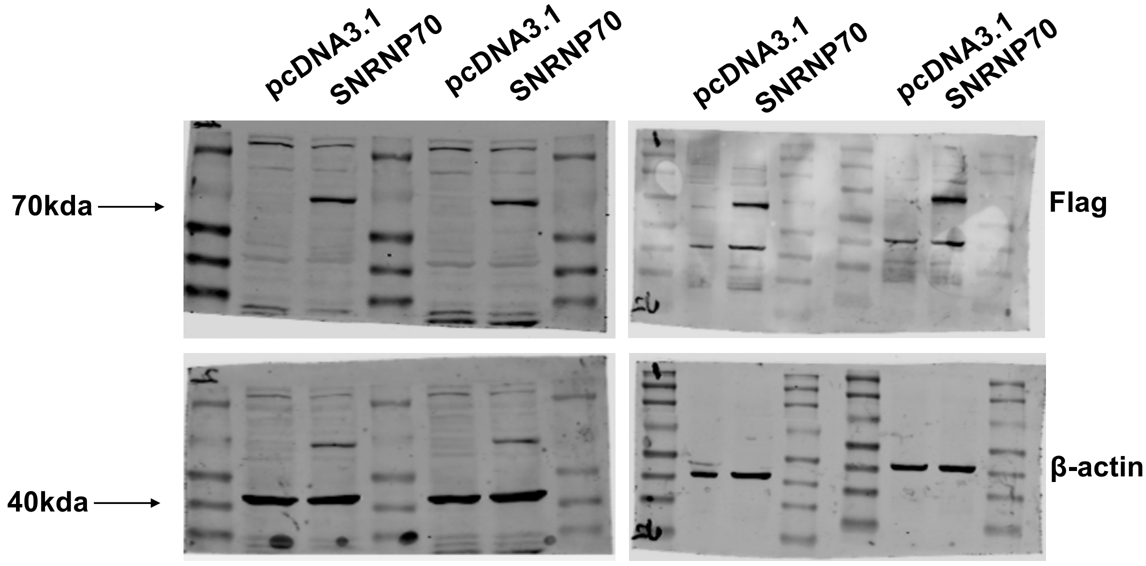

Fig.4A

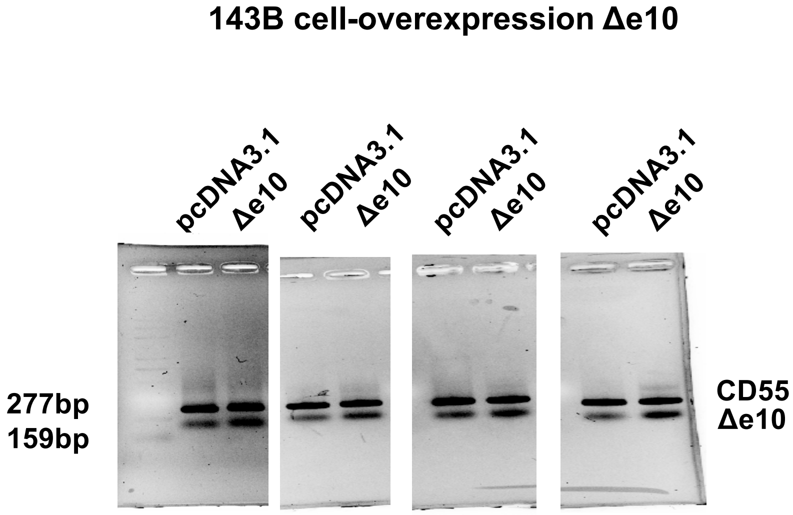

143B cell-overexpression Δe10
pcDNA3.1
Δe10
pcDNA3.1
Δe10
pcDNA3.1
Δe10
pcDNA3.1
Δe10
277bp
159bp
CD55
Δe10

Fig.4A

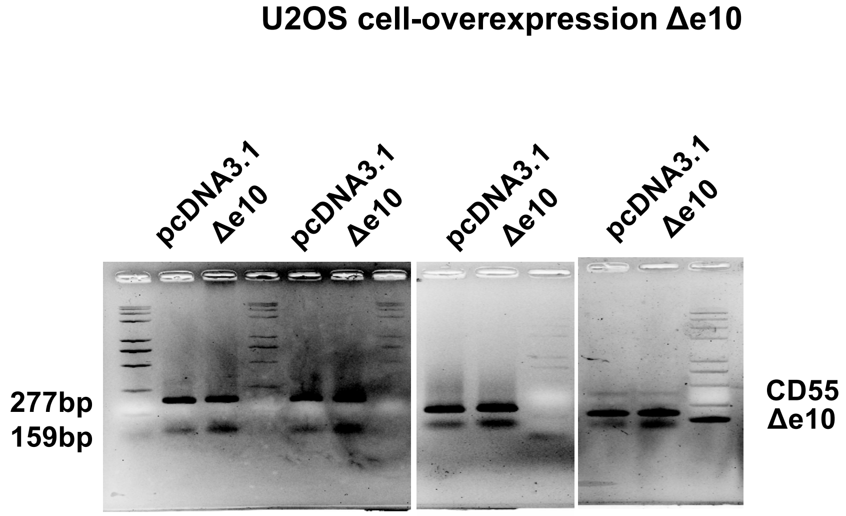
U2OS cell-overexpression Δe10
pcDNA3.1
Δe10
pcDNA3.1
Δe10
pcDNA3.1
Δe10
pcDNA3.1
Δe10
277bp
159bp
CD55
Δe10

Fig.4G

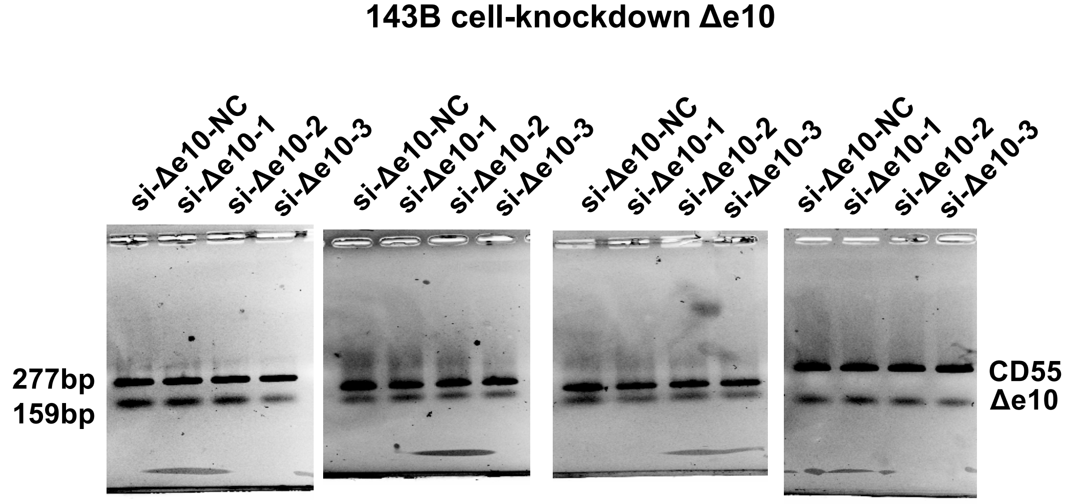
143B cell-knockdown Δe10
si-Δe10-NC
si-Δe10-1
si-Δe10-2
si-Δe10-3
si-Δe10-NC
si-Δe10-1
si-Δe10-2
si-Δe10-3
si-Δe10-NC
si-Δe10-1
si-Δe10-2
si-Δe10-3
si-Δe10-NC
si-Δe10-1
si-Δe10-2
si-Δe10-3
277bp
159bp
CD55
Δe10

Fig.4G

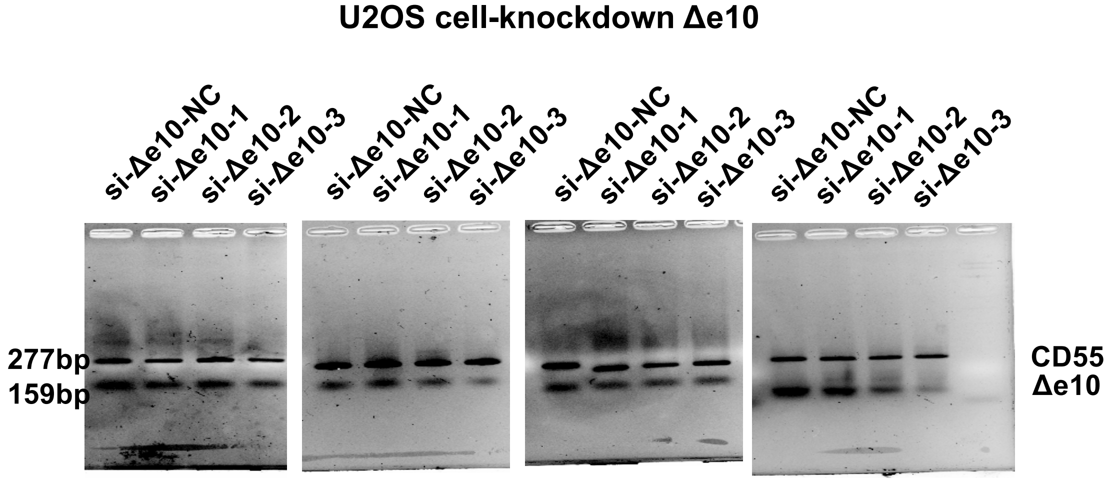
U2OS cell-knockdown Δe10
si-Δe10-NC
si-Δe10-1
si-Δe10-2
si-Δe10-3
si-Δe10-NC
si-Δe10-1
si-Δe10-2
si-Δe10-3
si-Δe10-NC
si-Δe10-1
si-Δe10-2
si-Δe10-3
si-Δe10-NC
si-Δe10-1
si-Δe10-2
si-Δe10-3
277bp
159bp
CD55
Δe10

Fig.6A

143B cell-knockdown SNRNP70(protein level)

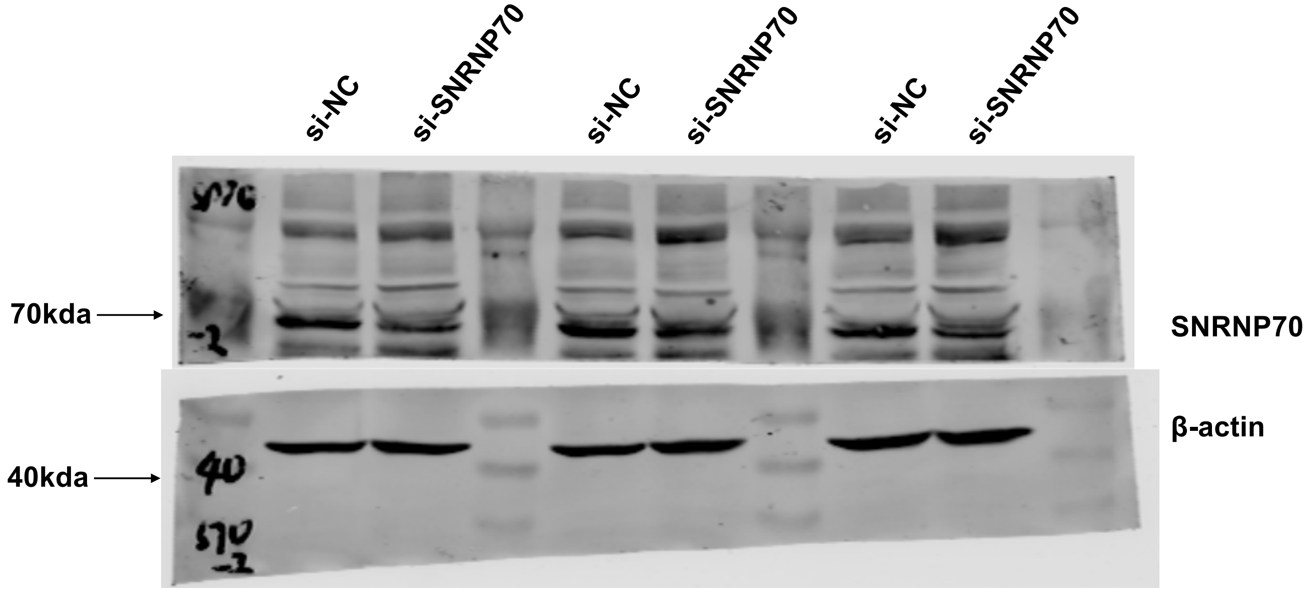

Supplement: Unedited blot and gel images [file jciinsight-9-185269-s147.pdf]
